# Supplementary material for: Physiologic effects of surgical masking in children versus adults
Source: PeerJ. 2023 Jun 16;11:e15474. doi: 10.7717/peerj.15474 (PMC10278594; doi:10.7717/peerj.15474)
Supplement: Supplemental Information 3 [file peerj-11-15474-s003.pdf]

Date: 1-31-2023

IRB #: IRB-FY2022-36
Title: The Effect of Wearing Surgical Mask on End-tidal Carbon Dioxide and Pulse Oximetry.
Creation Date: 7-26-2021
End Date:
Status: Approved
Principal Investigator: Patrick Brooks
Review Board: MSU
Sponsor:

Study History

Submission Type	Review Type	Decision
Initial	Expedited	Approved
Modification	Expedited	Approved

Key Study Contacts

Member	Role	Contact
Jill Layman	Co-Principal Investigator	jilllayman@missouristate.edu
Patrick Brooks	Principal Investigator	PatrickBrooks@missouristate.edu
Patrick Brooks	Primary Contact	PatrickBrooks@missouristate.edu
Caleb Dodd	Investigator	cpd502@live.missouristate.edu
Kayla Kline	Investigator	kayla64@live.missouristate.edu
Reagan Stange	Investigator	stange0820@live.missouristate.edu
Kaity Kuhnert	Investigator	kuhnert00@live.missouristate.edu

Member	Role	Contact
Kaitlyn Miller	Investigator	kpm773s@missouristate.edu
Krusha Bhakta	Investigator	krusha000@live.missouristate.edu
Breanna Skinner	Investigator	bs47s@missouristate.edu
Carla Castaneda Samillan	Investigator	carla521@live.missouristate.edu
Glory Ehie	Investigator	glo278@live.missouristate.edu
Ashlyn Spinabella	Investigator	spinabella125@live.missouristate.edu
Ashlynn Harmon	Investigator	abh9070@live.missouristate.edu

Initial Submission

Investigative Team

*required

Who is the Principal Investigator?

1 *This individual will be required to certify the protocol for submission and will be responsible for the overall project and **MUST be a faculty or staff** member.*

Name: Patrick Brooks
Organization: Biomedical Sciences
Address: 901 S National Avenue , Springfield, MO 65897-0027
Phone: 417-836-5603
Email: PatrickBrooks@missouristate.edu

*required

Who is the Primary Study Contact?

2 *This person, in addition to the Principal Investigator, will be included on all correspondence related to this project. This person may be the Principal Investigator or someone else **(faculty, staff, or student)**.*

Name: Patrick Brooks
Organization: Biomedical Sciences
Address: 901 S National Avenue , Springfield, MO 65897-0027
Phone: 417-836-5603
Email: PatrickBrooks@missouristate.edu

*required

Will there be any Co-Principal Investigators participating in this study?

3.

Co-Principal Investigators will also be required to certify the protocol for submission and share overall responsibility with the Principal Investigator for the study. Co-Principal Investigators MUST be faculty or staff members.

✓ Yes

*required

Select the Co-Principal Investigator(s).

Name: Jill Layman
Organization: School of Anesthesia
Address: 901 S. National Ave. , Springfield, MO 65897-0027
Phone:
Email: jilllayman@missouristate.edu

No

*required

Will there be any other individuals participating with the investigation?

4

These individuals will be participating as part of the research team, but will not need to certify the protocol submissions, or be included in any correspondence regarding the study. Typically these individuals will be students or individuals from other institutions. Investigators may be faculty, staff, students, or unaffiliated individuals.

Yes

✓ No

General Information

*required

1 What is the full title of the research protocol?

End-Tidal Carbon Dioxide Measurements in Adults and Children Wearing Surgical Masks

*required

Abstract/Summary

Please provide a brief description of the project.

In efforts to reduce the spread of the coronavirus, public health agencies, states & local governments, schools, and businesses have required individuals to wear masks. The public, and one retracted JAMA-Pediatrics research letter, have questioned the safety of wearing masks; specifically, the concerns about retained carbon dioxide in masked children. There is currently no medical or scientific concern about masks impairing carbon dioxide or oxygen exchange of the wearers, as evidenced by the American Academy of Pediatrics' recommendation for all children older than 2 years of age to wear masks in school this year.

2

Currently, minimal acceptable published studies measure the effect of mask-wearing on retained carbon dioxide, oxygen saturation, and heart rate. A Clinical Observational Study is planned to measure subjects' Carbon Dioxide levels using a non-invasive end-tidal carbon monoxide (ETCO2) monitor by way of a nasal canula (NC). Non-invasive pulse oximetry will measure oxygen saturation (SpO2). The respiratory rates and heart rates will also be measured.

Measurements will be taken without masks, then repeat measurements will be taken after wearing a mask for 15 minutes. Data will be collected from adults and children as young as 2 years of age. Age groups will include children aged 2-14 and adults aged 18 to 80, as described in the details of the research protocol. Parents and their children are invited to participate together.

*required

Are you requesting Single IRB Review

3

Single IRB Review is applicable to a study that is being reviewed by another Institution's IRB, in which you wish to rely on the external IRB for review, approval, and oversight.

Yes

✓ No

*required

Does the study require review and oversight of the IRB?

4

Regardless of how these questions are answered, the determination of IRB review and oversight is made by the IRB and this study will still need to be submitted for preliminary review.

*required

4A

Is this study a systematic investigation, following a predetermined plan, for looking at a particular issue, testing a hypothesis or research question, or developing a new theory that includes any of the following:

- Collection or analysis of quantitative or qualitative data
- Collection of data using surveys, testing or evaluation procedures, interviews, or focus groups
- Collection of data using experimental designs such as clinical trials
- Observation of individual or group behavior

✓ Yes

No

*required

4B

Will this study contribute to generalizable knowledge, in that the purpose or intent of the project is to test or to develop scientific theories or hypotheses, or to draw conclusions that are intended to be applicable and/or shared beyond the populations or situations being studied? This may include one or more of the following:

- Presentation of the data at meetings, conferences, seminars, poster presentations, etc.

- The knowledge contributes to an already established body of knowledge
- Other investigators, scholars, and practitioners may benefit from this knowledge
- Publications including journals, papers, dissertations, and theses

---

✓ Yes

No

*required

4C Will this study require obtaining information or biospecimens, through intervention or interaction with an individual that will be used, studied, or analyzed by the investigative team?

✓ Yes

No

*required

Will you be requesting an Exempt Review for this study?

5

In order to qualify for review via exempt procedures, the research must not be greater than minimal risk and must fall into at least one of the exempt categories defined by federal regulations.

Yes

✓ No

*required

6 Is this study receiving internal or external funding?

Yes

✓ No

*required

Does this study contain protected health information (PHI)?

7

PHI is any information in a medical record or designated record set that can be used to identify an individual and that was created, used, or disclosed in the course of providing a health care service, such as a diagnosis or treatment.

✓ Yes

*required

Please address all the HIPAA considerations/procedures of your study.

The following are examples of information that may need to be included, as applicable:

- *Procedures to be used regarding the Notice of Privacy Practices*
- *Procedures to be used in requesting Authorization for Use and Disclosure of PHI from Individuals*
- *Procedures to be used in requesting a waiver of Authorization from the IRB*
- *Statement that only de-identified individual health information will be used*
- *Request for access to PHI from a Health Care Component within Missouri State*
- *Request for access to PHI from a Covered Entity outside of Missouri State*

Researchers will be collecting measurements of the participants' end-tidal carbon dioxide, pulse oximetry, respiratory rate, and heart rate, at baseline, and at timed intervals after mask wearing.
Signatures and subject names will only be collected on the consent forms.
The ages of the subjects will be tracked and recorded with their physiologic measurements, in order to allow comparison of results with different age groups. Subject names will not be recorded with the ages.

No

*required

8 Has all IRB Human Research training been taken through CITI under Missouri State University?

✓ Yes

No

*required

Describe the proposed project in a manner that allows the IRB to gain a sense of the project including:

- The research questions and objectives,
- Key background literature (supportive and contradictory) with references, and
- The manner in which the proposed project will improve the understanding of the chosen topic.

Research question: How are ETCO2, pulse oximetry, and heart rate affected after wearing a mask for 15 minutes?

Objective: To evaluate the level of retained CO2 while wearing masks and the potential physiological effects.

Carbon Dioxide (CO2) is eliminated from our bodies by exhalation (breathing out). If a mask were to impair breathing significantly, the CO2 level would rise. The CO2 level in our body can be estimated by measuring the End-Tidal CO2 level. This is a measurement of the CO2 in our exhaled breath, and it is a close approximation of the CO2 in our bodies. End-tidal CO2 can be measured through a simple nasal cannula without any harm to the subject.

The below references are included to show the following points:
Pediatricians recommend that children over the age of 2 wear masks to school this year. The Medical community is not concerned about masks causing any problems for children.

A poorly done study published in JAMA Pediatrics has caused public commotion about the elevated CO2 behind masks worn by children. This study was retracted, but the public misinformation damage has been great.

Adult studies show that mask wearing is safe, even among exercising adults and adults with chronic lung disease.

Only one study has evaluated End-Tidal CO2 in masked children, and it showed only a minor elevation from N95 masks.

Loose fitting surgical masks, as planned in our study, are similar to cloth masks so cloth masks will not be studied.

Loose fitting surgical masks allow more breathability than N95 masks, and are expected to be safe.

End-Tidal CO2, a close estimate of our body's CO2 level, can be measured with a simple nasal cannula attached to an End-Tidal CO2 monitor.

There are no studies of End-Tidal CO2 levels in children wearing surgical masks. Completion and publication of this study will provide evidence beneficial to Physicians, School Boards, Parents, and Children about the safety of wearing masks.

Key background literature:

1. https://services.aap.org/en/pages/2019-novel-coron...
The American Association of Pediatrics Covid-19 Guidance for Safe Schools (as of 7/18/21) has recommended:

- All eligible individuals should receive the COVID-19 vaccine.
  - It may become necessary for schools to collect COVID-19 vaccine information of staff and students and for schools to require COVID-19 vaccination for in-person learning.
  - Adequate and timely COVID-19 vaccination resources for the whole school community must be available and accessible.
- **All students older than 2 years and all school staff should wear face masks at school (unless medical or developmental conditions prohibit use).**
  - The AAP recommends universal masking in school at this time for the following reasons:
    - a significant portion of the student population is not eligible for vaccination
    - protection of unvaccinated students from COVID-19 and to reduce transmission
      - lack of a system to monitor vaccine status among students, teachers, and staff
    - potential difficulty in monitoring or enforcing mask policies for those who are not vaccinated; in the absence of schools being able to conduct this monitoring, universal masking is the best and most effective strategy to create consistent messages, expectations, enforcement, and compliance without the added burden of needing to monitor vaccination status
    - possibility of low vaccination uptake within the surrounding school community
    - continued concerns for variants that are more easily spread among children, adolescents, and adults

2. Walach H, Weikl R, Prentice J, Diemer A, Traindl H, Kappes A, Hockertz S. Experimental Assessment of Carbon Dioxide Content in Inhaled Air With or Without Face Masks in Healthy Children: A Randomized Clinical Trial. JAMA Pediatr. 2021 Jun 30:e212659. doi: 10.1001/jamapediatrics.2021.2659. Epub ahead of print. Retraction in: JAMA Pediatr. 2021 Jul 16;:e213252. PMID: 34190984; PMCID: PMC8246331.

This published research letter was retracted, but not until after it caused a great amount of attention from concerned citizens. The authors used a flawed research design: they measured Carbon Dioxide (CO2) levels in the small space behind the masks worn by children. They inappropriately then compared it to recommendations for ambient Carbon Dioxide levels (meaning when high levels of CO2 fill a room). They avoided pointing out that the very small volume of CO2 behind the mask would be tremendously diluted by inspiration of fresh air from the surrounding room. The more appropriate question is whether mask wearing will alter the CO2 level in the wearer, yet they did not

measure the children's CO2 levels which can be measured noninvasively with an End-Tidal CO2 monitor. They did not reference any prior studies of mask wearing subjects that showed safe levels of CO2 or Oxygen. The publication was quickly retracted, yet it has been used to demand that school boards prevent masking of children in schools.

A proper study needs to be published to refute this highly flawed report.

3. Minhas JS, Robinson T, Panerai R. PaCO2 measurement in cerebral haemodynamics: face mask or nasal cannulae? *Physiological measurement*. 2017;38(7):N101-N106. doi:10.1088/1361-6579/aa6f9f

This study reviews the measurements of End-Tidal CO2, which is used as a noninvasive analog for arterial CO2 levels. It compared measurements of end-tidal CO2 by using full face masks or using nasal cannulas. The results from the two measurements were similar. This is referenced to show that the measurement of end-tidal CO2 by nasal cannula, as planned in our study, is an accurate measurement technique.

4. Konda A, Prakash A, Moss GA, Schmoldt M, Grant GD, Guha S. Aerosol Filtration Efficiency of Common Fabrics Used in Respiratory Cloth Masks. ACS Nano. 2020 May 26;14(5):6339-6347. doi: 10.1021/acsnano.0c03252. Epub 2020 Apr 24. Erratum in: ACS Nano. 2020 Aug 25;14(8):10742-10743. PMID: 32329337; PMCID: PMC7185834.

According to this article, cotton is the most widely used material for cloth masks. Higher thread counts, such as 600 threads per inch (TPI), provide the best filtration efficiency. Both one and two layer 600 TPI cotton masks have similar filtration efficiency as surgical masks, and the same breathability as measured by pressure differential of 2.5 Pascals.

1 Our study will therefore standardize the masks and use surgical masks for all subjects. There is no need to study cloth masks as well, because they have similar effects when compared to surgical masks.

5. Shaw K, Butcher S, Ko J, Zello GA, Chilibeck PD. Wearing of Cloth or Disposable Surgical Face Masks has no Effect on Vigorous Exercise Performance in Healthy Individuals. *Int J Environ Res Public Health*. 2020;17(21):8110. Published 2020 Nov 3. doi:10.3390/ijerph17218110

“When expressed relative to peak exercise performance, no differences were evident between wearing or not wearing a mask for arterial oxygen saturation, tissue oxygenation index, rating of perceived exertion, or heart rate at any time during the exercise tests. Wearing a face mask during vigorous exercise had no discernable detrimental effect on blood or muscle oxygenation, and exercise performance in young, healthy participants.”

We do not plan to test mask wearing during exertion, but this study shows further evidence that

surgical face masks are not harmful, even during vigorous exercise. Yet, the study did not measure End-Tidal CO_2 levels and did not study children.

6. Samannan R, Holt G, Calderon-Candelario R, Mirsaeidi M, Campos M. Effect of Face Masks on Gas Exchange in Healthy Persons and Patients with Chronic Obstructive Pulmonary Disease. Ann Am Thorac Soc. 2021 Mar;18(3):541-544. doi: 10.1513/AnnalsATS.202007-812RL. PMID: 33003954; PMCID: PMC7919152.

“these data find that gas exchange is not significantly affected by the use of surgical mask, even in subjects with severe lung impairment….

"It is important to inform the public that the discomfort associated with mask use should not lead to unsubstantiated safety concerns as this may attenuate the application of a practice proved to improve public health. As growing evidence indicates that asymptomatic individuals can fuel the spread of COVID-19 (12), universal mask use needs to be vigorously enforced in community settings, particularly now that we are facing a pandemic with minimal proven therapeutic interventions. We believe our data will help mitigate fears about the health risks of surgical mask use and improve public confidence for more widespread acceptance and use.”

This study shows further evidence that surgical face masks do not cause harm to healthy adults or adults with chronic lung disease. The study did not include children. We will not be studying any subjects with chronic lung disease.

7. Eberhart M, Orthaber S, Kerbl R. The impact of face masks on children-A mini review. Acta Paediatr. 2021 Jun;110(6):1778-1783. doi: 10.1111/apa.15784. Epub 2021 Feb 21. PMID: 33533522; PMCID: PMC8014099.

The complete abstract of this very recent review is included below. It discusses the limited information about children wearing face masks.

Abstract
Aim: Face masks are essential during the COVID-19 pandemic, and the United Nations Children's Fund and the World Health Organization, recommend that they are used for children aged six years and older. However, parents are increasingly expressing concerns about whether these might be physically harmful. This mini review assessed the evidence.
Method: We conducted a narrative review on the effects of mask wearing on physiological variables in children, using PubMed, the Cochrane Library and the World Health Organization COVID-19 Database up to 7 November 2020. The lack of paediatric studies prompted a second search for adult studies.
Results: We only found two paediatric studies, published in 2019 and 2020. The 2020 study was not related to COVID-19. Only one study, performed with N95 respirators, collected medical parameters, and this did not suggest any harmful effects of gas exchange. The eight adult studies, including four prompted by the pandemic and one on surgeons, reported that face masks commonly used during the pandemic did not impair gas exchange during rest or mild exercise.

Conclusion: International guidelines recommend face masks for children aged six years and older, but further studies are needed to provide evidence-based recommendations for different age groups

8. Goh DYT, Mun MW, Lee WLJ, Teoh OH, Rajgor DD. A randomised clinical trial to evaluate the safety, fit, comfort of a novel N95 mask in children. Sci Rep. 2019 Dec 12;9(1):18952. doi: 10.1038/s41598-019-55451-w. PMID: 31831801; PMCID: PMC6908682.

This is the only article found (confirmed by the above review by Eberhart) that measured End-Tidal CO2 levels in children wearing masks. It tested a novel N95 mask, not a surgical mask. There were small but not significant rises in End-Tidal C02 measurements after wearing the N95 mask. This particular model had a microfan that when activated lowered the CO2 levels back to baseline. As shown in the article below, a "loose fitting surgical mask", as planned in our study, has much higher breathability than an N95 mask.

9. Karuppasamy K, Obuchowski N. Comparison of Fit for Sealed and Loose-Fitting Surgical Masks and N95 Filtering Facepiece Respirators. Ann Work Expo Health. 2021 May 3;65(4):463-474. doi: 10.1093/annweh/wxaa125. PMID: 33458738; PMCID: PMC7929389.

In short, "loose fitting" surgical masks are planned in our study. This implies that the masks are not taped to the face along the edges. Loose fitting surgical masks in this study allow a much greater amount of air (and aerosols) to be breathed in during inspiration. (They are not ideal for high risk Covid contact, as in the Intensive Care Unit). N95 masks have the least amount of air leak during inspiration. Even a taped (sealed) surgical mask has more breathability and comfort than an N95 mask.

This study is mentioned in reference to the Goh article above. Although that article showed a mild but not significant increase in CO2 from wearing an N95 mask, our study with loose fitting surgical masks is expected to cause even less of an effect.

10. https://www.fauquier.com/opinion/letter-to-mask-or...

In this opinion letter to Fauquier Times in Virginia, the authors state:
"Armed with both the state of emergency expiration, as well as a peer-reviewed medical study stating that we, as decision makers, should not be forcing our children to wear masks, Fauquier County Public Schools put out guidance that masks shall be optional going forward. Each family is encouraged to examine their individual situation, weigh their personal risks against benefits, and make a decision that best suits their needs.
Yesterday, the state health commissioner released another executive order mandating masks for school-aged children.
No locality should be stuck trying to weigh a state mandate against a peer reviewed medical study, but here we are."

In summary:
To our knowledge, ours will be the first study measuring the End-Tidal CO2 levels in children wearing surgical masks. We will include adults in the study as well. This will provide an assessment of a wide age group of adults and children, and the parents are able to participate with their children. Completion and publication of this study will provide evidence beneficial to Physicians, School Boards, Parents, and Children about the safety of wearing surgical masks.

*required

2 Check all research activities that apply:

Audio, video, digital, or image recordings

Biohazards (e.g., rDNA, infectious agents, select agents, toxins)

✓ Biological sampling (other than blood)

Blood drawing

Class Protocol (or Program or Umbrella Protocol)

✓ Data, not publicly available

Data, publicly available

Deception

✓ Devices

Diet, exercise, or sleep modifications

Drugs or biologics

Focus groups

Intenet or email data collection

Materials that may be considered sensitive, offensive, threatening, or degrading

✓ Non-invasive medical procedures

Observation of participants

✓ Oral history

Placebo

Record review

Specimen research

Surgical procedures

Surveys, questionnaires, or interviews (one-on-one)

Surveys, questionnaires, or interviews (group)

Other

*required

Describe the procedures and methods planned for carrying out the study. Make sure to include the following:

- Site selection,
- The procedures used to gain permission to carry out research at the selected sites(s),
- Data collection procedures, and
- An overview of the manner in which data will be analyzed.

Provide all information necessary for the IRB to be clear about all of the contact human participants will have with the project.

Participants will be invited to Missouri State School of Anesthesia, where the trial will be held. Dr. Monika Feeney, Director of the School of Anesthesia, has approved the study and the use of the building and the anesthesia equipment; see attachment below. The anesthesia machines (2) will be calibrated before the study.

Adult subjects with children will be recruited by email and posters.

Adult Inclusion criteria: Ages 18 to 80 (inclusive) and agreeable to provide consent
Pediatric Inclusion criteria: Ages 2 to 14 years (inclusive) and parents are agreeable to provide consent. The lower age limit was chosen based on AAP recommendations, yet young participants in particular may not wish to keep their masks in place--they can exit the study at any time.

Exclusion criteria: The subjects fulfilling any of the exclusion criteria listed below will be excluded

1. any known cardiorespiratory conditions (including but not limited to the following: asthma, bronchitis, cystic fibrosis, congenital heart disease, adult heart disease, emphysema)
2. symptoms or concern for a respiratory tract infection or symptomatic rhinitis (i.e. blocked nasal passages, runny nose, significant sneezing, shortness of breath, new cough, symptomatic fever) on the day of the study
3. conditions that may compromise mask fit (e.g. excessive facial hair or craniofacial abnormalities)

A Physician , Nurse Anesthetist, or Nurse will briefly interview each participate to ensure they meet inclusion criteria and do not have exclusion criteria. Any subject can choose not to answer any questions and can avoid joining the study. Subjects can also withdraw from the study at any time. Participants will be assigned a number upon arrival, indicated by a sticker to be worn during the trial. No personal identifying information will be collected.

Trained assistants will apply the appropriate sized nasal cannula (NC) with a built-in ETCO2 monitoring port. Baseline NC-ETCO2, pulse oximetry (SpO2), respiratory rate (RR) and heart rate (HR) will be measured and documented. After the baseline is recorded, assistants will help participants apply a 'surgical' facemask. Participants will wear the mask for 15 minutes, at rest. Researchers will repeat NC-ETCO2, SpO2, and HR measurements. Data will be entered into Excel spreadsheets, including the assigned participants' numbers and the pre and post-NC ETCO2, SpO2, RR and HR. MSU RStats will analyze the data and results.

4 Attach tests, surveys, questionnaires, and other social-behavioral measurement tools, if applicable.

---

5 Attach documentation of site permission, if applicable.

Building and equipment permission.pdf

Participants

*required

1 Specify the participant population(s).

Check all that apply.

✓ Adults

✓ Children (<18 years of age)

Adults with decisional impairment

Non-English speaking

Student research pools (e.g. psychology)

Pregnant women or fetuses

Prisoners

Unknown (e.g., secondary use of data/specimens, non-targeted surveys, program/class/umbrella protocols)

*required

2 Specify the age(s) of the individuals who may participate in the research.

Ages 2-14 (inclusive) and 18-80 (inclusive) are invited to enroll in the study. Parents will remain with their children throughout the process.

*required

3 Describe the characteristics of the proposed participants, and explain how the nature of the research requires/justifies their inclusion.

Every age group has been affected by the recent pandemic and most age groups are included in the mask mandates occurring across our country. The American Association of Pediatricians recommends mask wearing for children age 2 and up as they attend school. Some have argued that

children should not have to wear masks due to the accumulation of carbon dioxide, however, schools and public spaces may require that masks be worn to mitigate the spread of the virus, particularly as more virulent and transmittable forms of the virus mutate. Because of the current rise in coronavirus cases in every state, the increase in the younger population affected, and the fact that the delta variant appears to be a virulent strain, we believe it is important to assess the effect of wearing a mask on all ages.

*required

4 Provide the total number of participants (or number of participant records, specimens, etc.) for whom you are seeking IRB approval.

---

80 - 500. Reviewing the power determination from the single pediatric study of End-Tidal CO2 levels in N95 masks (Goh et all, Scientific Reports, 2019), at least 80 subjects in the pediatric group are desired.

*required

5 Describe what time commitment will be required from each participant, including individual interactions, total time commitment, and long-term follow-up, if any.

The total participant time commitment will be approximately 1/2 hour. Because masks are being worn everywhere with no negative consequences, there will be no follow-up necessary.

*required

6 Describe how potential participants will be identified (e.g., advertising, individuals known to investigator, record review, etc.). Explain how investigator(s) will gain access to this population, as applicable.

Our study includes age groups from 2 to 14 (inclusive) and 18-80 (inclusive), individuals, and families. Researchers will invite those in the local community to participate in the study.

*required

7 Describe the recruitment process; including the setting in which recruitment will take place.

Recruitment of participants will occur via direct communication (word of mouth), emails, and flyers in the community.

Attach recruitment materials (ads, flyers, website postings, recruitment letters, and oral/written scripts), if applicable .

flyer mask CO2.pdf

*required

8 Will participants receive compensation or other incentives (e.g., free services, cash payments, gift certificates, parking, classroom credit, travel reimbursement, etc.) to participate in the research study?

Yes

✓ No

Risks and Benefits

*required

1 Describe all reasonably expected risks, harms, and/or discomforts that may apply to the research. Discuss severity and likelihood of occurrence.

Consider the range of risks - physical, psychological, social, legal, and economic.

There are no expected risks or harms related to this study. Although most participants have recently been required to wear masks in public, some may feel some discomfort in doing so. A new disposable surgical mask, of the same model, in appropriate sizes, will be worn by all participants. It is similar to one that is approved for the surgical theater and worn by all health care providers in an operating room. The nasal cannula, used daily in operating rooms in the US, is not associated with pain or discomfort but will be a new experience for participants.

*required

2 Discuss the steps that will be taken to minimize risks and the likelihood of harm.

Should a child or adult decline participation after masking or wearing a nasal cannula, they may withdraw from the study at any time.

*required

3 Describe the potential benefits that participants may expect as a result of this research study. State if there are no direct benefits to individual participants.

No direct benefits to individual participants.

*required

Discuss any potential indirect benefits to future subjects, science, and society.

The American Association of Pediatrics Covid-19 Guidance for Safe Schools (as of 7/18/21) has recommended:

- All eligible individuals should receive the COVID-19 vaccine.
    - It may become necessary for schools to collect COVID-19 vaccine information of staff and students and for schools to require COVID-19 vaccination for in-person learning.
    - Adequate and timely COVID-19 vaccination resources for the whole school community must be available and accessible.
- **All students older than 2 years and all school staff should wear face masks at school (unless medical or developmental conditions prohibit use).**
    - The AAP recommends universal masking in school at this time for the following reasons:
        - a significant portion of the student population is not eligible for vaccination
        - protection of unvaccinated students from COVID-19 and to reduce transmission
            - lack of a system to monitor vaccine status among students, teachers, and staff
        - potential difficulty in monitoring or enforcing mask policies for those who are not vaccinated; in the absence of schools being able to conduct this monitoring, universal masking is the best and most effective strategy to create consistent 4 messages, expectations, enforcement, and compliance without the added burden of needing to monitor vaccination status
        - possibility of low vaccination uptake within the surrounding school community
        - continued concerns for variants that are more easily spread among children, adolescents, and adults

Masking during the Covid-19 epidemic has been an emotionally and politically charged topic. There are widespread public misconceptions about the benefits and risks of masking, particularly in children. A since retracted JAMA Pediatrics publication claimed that masking was harmful to children due to elevated CO2 levels. As discussed elsewhere in this application, the study was fatally flawed and its conclusions were unsupported by scientific study or any appropriate scientific references. Nonetheless, that study sparked public outrage against mask use in schools and has been forwarded to school boards as "evidence" that masks should not be worn in schools.

This study, the first of its kind, aims to answer whether surgical masks, worn by both adults and children, cause any concerning abnormality in oxygenation or carbon dioxide levels, as measured by noninvasive means. Despite the fact that adults and children have been wearing masks for extended periods of time without low oxygen or high carbon dioxide side effects, there is an appetite for the final answer on this topic. School systems, parents, and the general public can benefit from seeing the results of this study.

*required

Describe how risks to participants are reasonable when compared to the anticipated benefits to participants (if any) and teh importance of the knowledge that may reasonably be expected to result.

Children and adults wear surgical-type masks without harm, as is evidenced by recommendations from the American Academy of Pediatricians. The novel experience of volunteer adults and children wearing a nasal cannula will be outweighed by their general interest in helping answer a sometimes emotionally charged question and providing knowledge to parents, educators, and the public.

Informed Consent

*required

1 From the list below, indicate how consent will be obtained for this study.

Check all that apply.

- ✓ Written/signed consent by the subject
- ✓ Written/signed consent (permission) for a minor by a Parent or Legal Guardian
- Written/signed consent by a Legally Authorized Representative (for adults incapable of consenting)
- Request for waiver of documentation of consent (verbal consent, anonymous surveys, etc.)
- Waiver of parental permission
- Waiver of consent (consent will not be obtained from subjects)

*required

2 Describe the consent process including where and by whom the subjects will be approached, the plans to ensure the privacy of the subjects and the measures to ensure that subjects understand the nature of the study, its procedures, risks and benefits and that they freely grant their consent.

Upon arrival to the designated area, participants will be instructed on the study, the procedures, any risks or benefits, and given the consent form for signature, for either adult or for a minor by a parent or legal guardian. The subjects/parents of subjects will speak with a medical professional with experience working with medical consent forms, and freely granted consent will be assured. All questions will be addressed. The subjects can choose to avoid answering the inclusion/exclusion screening questions and can avoid joining the study, or withdraw from the study, at any time.

*required

Attach all consent and assent documents here:

Assent Form Mask CO2 study.docx

consent CO2 mask study.docx

Missouri State University is committed to keeping data and information secure. Please review the Missouri Ste University Information Security Policies. Discuss you project with the MSU Information Security Office or your College's IT support staff if you have questions about how to handle your data appropriately.

*required

1 **Statement of Principal Investigator Responsibility for Data**

The principal investigator of this study is responsible for the storage, oversight, and disposal of all data associated with this study. Data will not be disseminated without the explicit approval of the principal investigator, and identifying information associated with the data will not be shared.

✓ By checking this box, all personnel associated with this study understand and agree to the Statement of Principal Investigator Responsibility for Data.

*required

2 How will the data for this study be collected/stored?

Check all that apply.

✓ Electronic Storage Format

On paper

*required

Describe where the data will be stored (e.g., paper forms, flash drives or removable media, desktop or laptop computer, server, research storage area network, external source) and describe the plan to ensure the security and confidentiality of the records (e.g., locked office, locked file cabinet, password-protected computer or files, encrypted data files, database limited to coded data, master list stored in separate location).

---

3 *At minimum, physical data should always be secured by lock and key when stored. Electronic data should be stored on University secure servers whenever possible (Office 365 or other secure campus server). If data has to be stored off campus, the file should be encrypted and the device password protected. Additionally, any data to be shared outside the University network will require a SUDERS request be filed and approved. See https://mis.missouristate.edu/Central/suders/create*

Data will be stored on an excel spreadsheet on the Missouri State onedrive. The excel spreadsheet will list the subjects only by subject number. No identifying data will be included on the spreadsheet. The study consent forms will be the only location which includes patients' names. These will be kept in a locked cabinet in the Department of Anesthesia.

*required

Describe how data will be disposed of and when disposal will occur.

---

4 *At minimum, Federal regulations require research records to be retained for at least 3 years after the completion of the research (45 CFR 46). Research that involves identifiable health information is subject to HIPAA regulations, which require records to be retained for at least 6 years after a participant has signed an authorization. Finally, funded research projects may require longer retention periods, you may need to follow the sponsoring agency guidelines.*

Excel spreadsheets, which will not include identifyable data, will be deleted after 3 years.

Consent forms, which include patients names/signatures will be destroyed by shreading after 6 years.

Additional Information

1 Please include any additional information about the study below.

2 Please include any additional documents that aren't covered within the application.

Modification Submission

Modification Summary

Please make changes to the original protocol sections below. In addition, provide a summary of the changes by completing the questions on this page.

*required

A. To which of the following aspects of research does this modification request apply?

Check all that apply.

✔ Change in personnel

Please include the name of the researcher(s) added to section 1 and attach their CITI training certificates in section 9.

Research design

Risks to participants or others in relation to anticipated benefits

Participant selection or recruitment process

Consent process and/or compensation

Methods for documenting consent

Change in supporting documentation or attachments

Potential willingness of research participants to continue to take part in this study

Monitoring of the data being collected

Privacy of the research participants and/or confidentiality of research participants' data

Other

*required

B.

Please provide a brief rationale for each of the changes being requested.

Adding student researchers to the team.   Cayuse would not let me find any student names under the Investigative team.

The students researchers are:

Kayla Kline
Reagan Stange
Caleb Dodd
Kaity Kuhnert
Kaitlyn Miller
Krusha Bhakta
Breanna Skinner
Carla Castaneda-Samillian
Glory Ehie
Ashlyn Spinabella
Ashlynn Harmon

Investigative Team

*required

Who is the Principal Investigator?

1 *This individual will be required to certify the protocol for submission and will be responsible for the overall project and **MUST be a faculty or staff** member.*

Name: Patrick Brooks
Organization: Biomedical Sciences
Address: 901 S National Avenue , Springfield, MO 65897-0027
Phone: 417-836-5603
Email: PatrickBrooks@missouristate.edu

*required

Who is the Primary Study Contact?

2 *This person, in addition to the Principal Investigator, will be included on all correspondence related to this project. This person may be the Principal Investigator or someone else **(faculty, staff, or student)**.*

Name: Patrick Brooks
Organization: Biomedical Sciences
Address: 901 S National Avenue , Springfield, MO 65897-0027
Phone: 417-836-5603
Email: PatrickBrooks@missouristate.edu

*required

Will there be any Co-Principal Investigators participating in this study?

3.

Co-Principal Investigators will also be required to certify the protocol for submission and share overall responsibility with the Principal Investigator for the study. Co-Principal Investigators MUST be faculty or staff members.

✓ Yes

*required

Select the Co-Principal Investigator(s).

Name: Jill Layman
Organization: School of Anesthesia
Address: 901 S. National Ave. , Springfield, MO 65897-0027
Phone:
Email: jilllayman@missouristate.edu

No

*required

Will there be any other individuals participating with the investigation?

4

These individuals will be participating as part of the research team, but will not need to certify the protocol submissions, or be included in any correspondence regarding the study. Typically these individuals will be students or individuals from other institutions. Investigators may be faculty, staff, students, or unaffiliated individuals.

✓ Yes

*required

Select the Investigator(s)

Name: Caleb Dodd
Organization: Kinesiology
Address: , Springfield, MO 65897-0027
Phone:
Email: cpd502@live.missouristate.edu

Name: Kayla Kline
Organization: Biomedical Sciences
Address: , Springfield, MO 65897-0027
Phone:
Email: kayla64@live.missouristate.edu

Name: Reagan Stange
Organization: Biomedical Sciences
Address: , Springfield, MO 65897-0027
Phone:
Email: stange0820@live.missouristate.edu

Name: Kaity Kuhnert
Organization: Nursing
Address: , Springfield, MO 65897-0027
Phone:
Email: kuhnert00@live.missouristate.edu

Name: Kaitlyn Miller
Organization: Biomedical Sciences
Address: , Springfield, MO 65897-0027
Phone:
Email: kpm773s@missouristate.edu

Name: Krusha Bhakta
Organization: Chemistry
Address: , Springfield, MO 65897-0027
Phone:
Email: krusha000@live.missouristate.edu

Name: Breanna Skinner
Organization: Biomedical Sciences
Address: , Springfield, MO 65897-0027
Phone:
Email: bs47s@missouristate.edu

Name: Carla Castaneda Samillan
Organization: Chemistry
Address: , Springfield, MO 65897-0027
Phone:
Email: carla521@live.missouristate.edu

Name: Glory Ehie
Organization: Biology
Address: , Springfield, MO 65897-0027
Phone:
Email: glo278@live.missouristate.edu

Name: Ashlyn Spinabella
Organization: Biomedical Sciences
Address: , Springfield, MO 65897-0027
Phone:
Email: spinabella125@live.missouristate.edu

Name: Ashlynn Harmon
Organization: Users loaded with unmatched Organization affiliation.
Address: , Springfield, MO 65897-0027
Phone:
Email: abh9070@live.missouristate.edu

No

General Information

*required

1 What is the full title of the research protocol?

End-Tidal Carbon Dioxide Measurements in Adults and Children Wearing Surgical Masks

*required

Abstract/Summary

Please provide a brief description of the project.

In efforts to reduce the spread of the coronavirus, public health agencies, states & local governments, schools, and businesses have required individuals to wear masks. The public, and one retracted JAMA-Pediatrics research letter, have questioned the safety of wearing masks; specifically, the concerns about retained carbon dioxide in masked children. There is currently no medical or scientific concern about masks impairing carbon dioxide or oxygen exchange of the wearers, as evidenced by the American Academy of Pediatrics' recommendation for all children older than 2 years of age to wear masks in school this year.

2

Currently, minimal acceptable published studies measure the effect of mask-wearing on retained carbon dioxide, oxygen saturation, and heart rate. A Clinical Observational Study is planned to measure subjects' Carbon Dioxide levels using a non-invasive end-tidal carbon monoxide (ETCO2) monitor by way of a nasal canula (NC). Non-invasive pulse oximetry will measure oxygen saturation (SpO2). The respiratory rates and heart rates will also be measured.

Measurements will be taken without masks, then repeat measurements will be taken after wearing a mask for 15 minutes. Data will be collected from adults and children as young as 2 years of age. Age groups will include children aged 2-14 and adults aged 18 to 80, as described in the details of the research protocol. Parents and their children are invited to participate together.

*required

Are you requesting Single IRB Review

3

Single IRB Review is applicable to a study that is being reviewed by another Institution's IRB, in which you wish to rely on the external IRB for review, approval, and oversight.

Yes

✓ No

*required

Does the study require review and oversight of the IRB?

4

Regardless of how these questions are answered, the determination of IRB review and oversight is made by the IRB and this study will still need to be submitted for preliminary review.

*required

4A

Is this study a systematic investigation, following a predetermined plan, for looking at a particular issue, testing a hypothesis or research question, or developing a new theory that includes any of the following:

- Collection or analysis of quantitative or qualitative data
- Collection of data using surveys, testing or evaluation procedures, interviews, or focus groups
- Collection of data using experimental designs such as clinical trials
- Observation of individual or group behavior

✓ Yes

No

*required

4B

Will this study contribute to generalizable knowledge, in that the purpose or intent of the project is to test or to develop scientific theories or hypotheses, or to draw conclusions that are intended to be applicable and/or shared beyond the populations or situations being studied? This may include one or more of the following:

- Presentation of the data at meetings, conferences, seminars, poster presentations, etc.

- The knowledge contributes to an already established body of knowledge
- Other investigators, scholars, and practitioners may benefit from this knowledge
- Publications including journals, papers, dissertations, and theses

✓ Yes

No

*required

4C Will this study require obtaining information or biospecimens, through intervention or interaction with an individual that will be used, studied, or analyzed by the investigative team?

✓ Yes

No

*required

5 Will you be requesting an Exempt Review for this study?

In order to qualify for review via exempt procedures, the research must not be greater than minimal risk and must fall into at least one of the exempt categories defined by federal regulations.

Yes

✓ No

*required

6 Is this study receiving internal or external funding?

Yes

✓ No

*required

Does this study contain protected health information (PHI)?

7

PHI is any information in a medical record or designated record set that can be used to identify an individual and that was created, used, or disclosed in the course of providing a health care service, such as a diagnosis or treatment.

✓ Yes

*required

Please address all the HIPAA considerations/procedures of your study.

The following are examples of information that may need to be included, as applicable:

- *Procedures to be used regarding the Notice of Privacy Practices*
- *Procedures to be used in requesting Authorization for Use and Disclosure of PHI from Individuals*
- *Procedures to be used in requesting a waiver of Authorization from the IRB*
- *Statement that only de-identified individual health information will be used*
- *Request for access to PHI from a Health Care Component within Missouri State*
- *Request for access to PHI from a Covered Entity outside of Missouri State*

Researchers will be collecting measurements of the participants' end-tidal carbon dioxide, pulse oximetry, respiratory rate, and heart rate, at baseline, and at timed intervals after mask wearing.
Signatures and subject names will only be collected on the consent forms.
The ages of the subjects will be tracked and recorded with their physiologic measurements, in order to allow comparison of results with different age groups. Subject names will not be recorded with the ages.

No

*required

8 Has all IRB Human Research training been taken through CITI under Missouri State University?

✓ Yes

No

*required

Describe the proposed project in a manner that allows the IRB to gain a sense of the project including:

- The research questions and objectives,
- Key background literature (supportive and contradictory) with references, and
- The manner in which the proposed project will improve the understanding of the chosen topic.

Research question: How are ETCO2, pulse oximetry, and heart rate affected after wearing a mask for 15 minutes?

Objective: To evaluate the level of retained CO_2 while wearing masks and the potential physiological effects.

Carbon Dioxide (CO_2) is eliminated from our bodies by exhalation (breathing out). If a mask were to impair breathing significantly, the CO_2 level would rise. The CO_2 level in our body can be estimated by measuring the End-Tidal CO_2 level. This is a measurement of the CO_2 in our exhaled breath, and it is a close approximation of the CO_2 in our bodies. End-tidal CO_2 can be measured through a simple nasal cannula without any harm to the subject.

The below references are included to show the following points:
Pediatricians recommend that children over the age of 2 wear masks to school this year. The Medical community is not concerned about masks causing any problems for children.

A poorly done study published in JAMA Pediatrics has caused public commotion about the elevated CO_2 behind masks worn by children. This study was retracted, but the public misinformation damage has been great.

Adult studies show that mask wearing is safe, even among exercising adults and adults with chronic lung disease.

Only one study has evaluated End-Tidal CO_2 in masked children, and it showed only a minor elevation from N95 masks.

Loose fitting surgical masks, as planned in our study, are similar to cloth masks so cloth masks will not be studied.

Loose fitting surgical masks allow more breathability than N95 masks, and are expected to be safe.

End-Tidal CO_2, a close estimate of our body's CO_2 level, can be measured with a simple nasal cannula attached to an End-Tidal CO_2 monitor.

There are no studies of End-Tidal CO_2 levels in children wearing surgical masks. Completion and publication of this study will provide evidence beneficial to Physicians, School Boards, Parents, and Children about the safety of wearing masks.

Key background literature:

1. https://services.aap.org/en/pages/2019-novel-coron...

The American Association of Pediatrics Covid-19 Guidance for Safe Schools (as of 7/18/21) has recommended:

- All eligible individuals should receive the COVID-19 vaccine.
  - It may become necessary for schools to collect COVID-19 vaccine information of staff and students and for schools to require COVID-19 vaccination for in-person learning.
  - Adequate and timely COVID-19 vaccination resources for the whole school community must be available and accessible.
- **All students older than 2 years and all school staff should wear face masks at school (unless medical or developmental conditions prohibit use).**
  - The AAP recommends universal masking in school at this time for the following reasons:
    - a significant portion of the student population is not eligible for vaccination
    - protection of unvaccinated students from COVID-19 and to reduce transmission
      - lack of a system to monitor vaccine status among students, teachers, and staff
    - potential difficulty in monitoring or enforcing mask policies for those who are not vaccinated; in the absence of schools being able to conduct this monitoring, universal masking is the best and most effective strategy to create consistent messages, expectations, enforcement, and compliance without the added burden of needing to monitor vaccination status
    - possibility of low vaccination uptake within the surrounding school community
    - continued concerns for variants that are more easily spread among children, adolescents, and adults

2. Walach H, Weikl R, Prentice J, Diemer A, Traindl H, Kappes A, Hockertz S. Experimental Assessment of Carbon Dioxide Content in Inhaled Air With or Without Face Masks in Healthy Children: A Randomized Clinical Trial. JAMA Pediatr. 2021 Jun 30:e212659. doi: 10.1001/jamapediatrics.2021.2659. Epub ahead of print. Retraction in: JAMA Pediatr. 2021 Jul 16;:e213252. PMID: 34190984; PMCID: PMC8246331.

This published research letter was retracted, but not until after it caused a great amount of attention from concerned citizens. The authors used a flawed research design: they measured Carbon Dioxide (CO_2) levels in the small space behind the masks worn by children. They inappropriately then compared it to recommendations for ambient Carbon Dioxide levels (meaning when high levels of CO_2 fill a room). They avoided pointing out that the very small volume of CO_2 behind the mask would be tremendously diluted by inspiration of fresh air from the surrounding room. The more appropriate question is whether mask wearing will alter the CO_2 level in the wearer, yet they did not

measure the children's CO2 levels which can be measured noninvasively with an End-Tidal CO2 monitor. They did not reference any prior studies of mask wearing subjects that showed safe levels of CO2 or Oxygen. The publication was quickly retracted, yet it has been used to demand that school boards prevent masking of children in schools.

A proper study needs to be published to refute this highly flawed report.

3. Minhas JS, Robinson T, Panerai R. PaCO2 measurement in cerebral haemodynamics: face mask or nasal cannulae? *Physiological measurement*. 2017;38(7):N101-N106. doi:10.1088/1361-6579/aa6f9f

This study reviews the measurements of End-Tidal CO2, which is used as a noninvasive analog for arterial CO2 levels. It compared measurements of end-tidal CO2 by using full face masks or using nasal cannulas. The results from the two measurements were similar. This is referenced to show that the measurement of end-tidal CO2 by nasal cannula, as planned in our study, is an accurate measurement technique.

4. Konda A, Prakash A, Moss GA, Schmoldt M, Grant GD, Guha S. Aerosol Filtration Efficiency of Common Fabrics Used in Respiratory Cloth Masks. ACS Nano. 2020 May 26;14(5):6339-6347. doi: 10.1021/acsnano.0c03252. Epub 2020 Apr 24. Erratum in: ACS Nano. 2020 Aug 25;14(8):10742-10743. PMID: 32329337; PMCID: PMC7185834.

According to this article, cotton is the most widely used material for cloth masks. Higher thread counts, such as 600 threads per inch (TPI), provide the best filtration efficiency. Both one and two layer 600 TPI cotton masks have similar filtration efficiency as surgical masks, and the same breathability as measured by pressure differential of 2.5 Pascals.

1 Our study will therefore standardize the masks and use surgical masks for all subjects. There is no need to study cloth masks as well, because they have similar effects when compared to surgical masks.

5. Shaw K, Butcher S, Ko J, Zello GA, Chilibeck PD. Wearing of Cloth or Disposable Surgical Face Masks has no Effect on Vigorous Exercise Performance in Healthy Individuals. *Int J Environ Res Public Health*. 2020;17(21):8110. Published 2020 Nov 3. doi:10.3390/ijerph17218110

“When expressed relative to peak exercise performance, no differences were evident between wearing or not wearing a mask for arterial oxygen saturation, tissue oxygenation index, rating of perceived exertion, or heart rate at any time during the exercise tests. Wearing a face mask during vigorous exercise had no discernable detrimental effect on blood or muscle oxygenation, and exercise performance in young, healthy participants.”

We do not plan to test mask wearing during exertion, but this study shows further evidence that

surgical face masks are not harmful, even during vigorous exercise. Yet, the study did not measure End-Tidal CO2 levels and did not study children.

6. Samannan R, Holt G, Calderon-Candelario R, Mirsaeidi M, Campos M. Effect of Face Masks on Gas Exchange in Healthy Persons and Patients with Chronic Obstructive Pulmonary Disease. Ann Am Thorac Soc. 2021 Mar;18(3):541-544. doi: 10.1513/AnnalsATS.202007-812RL. PMID: 33003954; PMCID: PMC7919152.

“these data find that gas exchange is not significantly affected by the use of surgical mask, even in subjects with severe lung impairment….

"It is important to inform the public that the discomfort associated with mask use should not lead to unsubstantiated safety concerns as this may attenuate the application of a practice proved to improve public health. As growing evidence indicates that asymptomatic individuals can fuel the spread of COVID-19 (12), universal mask use needs to be vigorously enforced in community settings, particularly now that we are facing a pandemic with minimal proven therapeutic interventions. We believe our data will help mitigate fears about the health risks of surgical mask use and improve public confidence for more widespread acceptance and use.”

This study shows further evidence that surgical face masks do not cause harm to healthy adults or adults with chronic lung disease. The study did not include children. We will not be studying any subjects with chronic lung disease.

7. Eberhart M, Orthaber S, Kerbl R. The impact of face masks on children-A mini review. Acta Paediatr. 2021 Jun;110(6):1778-1783. doi: 10.1111/apa.15784. Epub 2021 Feb 21. PMID: 33533522; PMCID: PMC8014099.

The complete abstract of this very recent review is included below. It discusses the limited information about children wearing face masks.

Abstract
Aim: Face masks are essential during the COVID-19 pandemic, and the United Nations Children's Fund and the World Health Organization, recommend that they are used for children aged six years and older. However, parents are increasingly expressing concerns about whether these might be physically harmful. This mini review assessed the evidence.
Method: We conducted a narrative review on the effects of mask wearing on physiological variables in children, using PubMed, the Cochrane Library and the World Health Organization COVID-19 Database up to 7 November 2020. The lack of paediatric studies prompted a second search for adult studies.
Results: We only found two paediatric studies, published in 2019 and 2020. The 2020 study was not related to COVID-19. Only one study, performed with N95 respirators, collected medical parameters, and this did not suggest any harmful effects of gas exchange. The eight adult studies, including four prompted by the pandemic and one on surgeons, reported that face masks commonly used during the pandemic did not impair gas exchange during rest or mild exercise.

Conclusion: International guidelines recommend face masks for children aged six years and older, but further studies are needed to provide evidence-based recommendations for different age groups

8. Goh DYT, Mun MW, Lee WLJ, Teoh OH, Rajgor DD. A randomised clinical trial to evaluate the safety, fit, comfort of a novel N95 mask in children. Sci Rep. 2019 Dec 12;9(1):18952. doi: 10.1038/s41598-019-55451-w. PMID: 31831801; PMCID: PMC6908682.

This is the only article found (confirmed by the above review by Eberhart) that measured End-Tidal CO2 levels in children wearing masks. It tested a novel N95 mask, not a surgical mask. There were small but not significant rises in End-Tidal C02 measurements after wearing the N95 mask. This particular model had a microfan that when activated lowered the CO2 levels back to baseline. As shown in the article below, a "loose fitting surgical mask", as planned in our study, has much higher breathability than an N95 mask.

9. Karuppasamy K, Obuchowski N. Comparison of Fit for Sealed and Loose-Fitting Surgical Masks and N95 Filtering Facepiece Respirators. Ann Work Expo Health. 2021 May 3;65(4):463-474. doi: 10.1093/annweh/wxaa125. PMID: 33458738; PMCID: PMC7929389.

In short, "loose fitting" surgical masks are planned in our study. This implies that the masks are not taped to the face along the edges. Loose fitting surgical masks in this study allow a much greater amount of air (and aerosols) to be breathed in during inspiration. (They are not ideal for high risk Covid contact, as in the Intensive Care Unit). N95 masks have the least amount of air leak during inspiration. Even a taped (sealed) surgical mask has more breathability and comfort than an N95 mask.

This study is mentioned in reference to the Goh article above. Although that article showed a mild but not significant increase in CO2 from wearing an N95 mask, our study with loose fitting surgical masks is expected to cause even less of an effect.

10. https://www.fauquier.com/opinion/letter-to-mask-or...

In this opinion letter to Fauquier Times in Virginia, the authors state:
"Armed with both the state of emergency expiration, as well as a peer-reviewed medical study stating that we, as decision makers, should not be forcing our children to wear masks, Fauquier County Public Schools put out guidance that masks shall be optional going forward. Each family is encouraged to examine their individual situation, weigh their personal risks against benefits, and make a decision that best suits their needs.
Yesterday, the state health commissioner released another executive order mandating masks for school-aged children.
No locality should be stuck trying to weigh a state mandate against a peer reviewed medical study, but here we are."

In summary:
To our knowledge, ours will be the first study measuring the End-Tidal CO_2 levels in children wearing surgical masks. We will include adults in the study as well. This will provide an assessment of a wide age group of adults and children, and the parents are able to participate with their children. Completion and publication of this study will provide evidence beneficial to Physicians, School Boards, Parents, and Children about the safety of wearing surgical masks.

*required

2 Check all research activities that apply:

Audio, video, digital, or image recordings

Biohazards (e.g., rDNA, infectious agents, select agents, toxins)

✓ Biological sampling (other than blood)

Blood drawing

Class Protocol (or Program or Umbrella Protocol)

✓ Data, not publicly available

Data, publicly available

Deception

✓ Devices

Diet, exercise, or sleep modifications

Drugs or biologics

Focus groups

Intenet or email data collection

Materials that may be considered sensitive, offensive, threatening, or degrading

✓ Non-invasive medical procedures

Observation of participants

✓ Oral history

Placebo

Record review

Specimen research

Surgical procedures

Surveys, questionnaires, or interviews (one-on-one)

Surveys, questionnaires, or interviews (group)

Other

*required

Describe the procedures and methods planned for carrying out the study. Make sure to include the following:

- Site selection,
- The procedures used to gain permission to carry out research at the selected sites(s),
- Data collection procedures, and
- An overview of the manner in which data will be analyzed.

Provide all information necessary for the IRB to be clear about all of the contact human participants will have with the project.

Participants will be invited to Missouri State School of Anesthesia, where the trial will be held. Dr. Monika Feeney, Director of the School of Anesthesia, has approved the study and the use of the building and the anesthesia equipment; see attachment below. The anesthesia machines (2) will be calibrated before the study.

Adult subjects with children will be recruited by email and posters.

Adult Inclusion criteria: Ages 18 to 80 (inclusive) and agreeable to provide consent
3 **Pediatric Inclusion criteria:** Ages 2 to 14 years (inclusive) and parents are agreeable to provide consent. The lower age limit was chosen based on AAP recommendations, yet young participants in particular may not wish to keep their masks in place--they can exit the study at any time.

Exclusion criteria: The subjects fulfilling any of the exclusion criteria listed below will be excluded

1. any known cardiorespiratory conditions (including but not limited to the following: asthma, bronchitis, cystic fibrosis, congenital heart disease, adult heart disease, emphysema)
2. symptoms or concern for a respiratory tract infection or symptomatic rhinitis (i.e. blocked nasal passages, runny nose, significant sneezing, shortness of breath, new cough, symptomatic fever) on the day of the study
3. conditions that may compromise mask fit (e.g. excessive facial hair or craniofacial abnormalities)

A Physician , Nurse Anesthetist, or Nurse will briefly interview each participate to ensure they meet inclusion criteria and do not have exclusion criteria. Any subject can choose not to answer any questions and can avoid joining the study. Subjects can also withdraw from the study at any time. Participants will be assigned a number upon arrival, indicated by a sticker to be worn during the trial. No personal identifying information will be collected.

Trained assistants will apply the appropriate sized nasal cannula (NC) with a built-in ETCO2 monitoring port. Baseline NC-ETCO2, pulse oximetry (SpO2), respiratory rate (RR) and heart rate (HR) will be measured and documented. After the baseline is recorded, assistants will help participants apply a 'surgical' facemask. Participants will wear the mask for 15 minutes, at rest. Researchers will repeat NC-ETCO2, SpO2, and HR measurements. Data will be entered into Excel spreadsheets, including the assigned participants' numbers and the pre and post-NC ETCO2, SpO2, RR and HR. MSU RStats will analyze the data and results.

4 Attach tests, surveys, questionnaires, and other social-behavioral measurement tools, if applicable.

---

5 Attach documentation of site permission, if applicable.

Building and equipment permission.pdf

Participants

*required

1 Specify the participant population(s).

Check all that apply.

✓ Adults

✓ Children (<18 years of age)

Adults with decisional impairment

Non-English speaking

Student research pools (e.g. psychology)

Pregnant women or fetuses

Prisoners

Unknown (e.g., secondary use of data/specimens, non-targeted surveys, program/class/umbrella protocols)

*required

2 Specify the age(s) of the individuals who may participate in the research.

Ages 2-14 (inclusive) and 18-80 (inclusive) are invited to enroll in the study. Parents will remain with their children throughout the process.

*required

3 Describe the characteristics of the proposed participants, and explain how the nature of the research requires/justifies their inclusion.

Every age group has been affected by the recent pandemic and most age groups are included in the mask mandates occurring across our country. The American Association of Pediatricians recommends mask wearing for children age 2 and up as they attend school. Some have argued that

children should not have to wear masks due to the accumulation of carbon dioxide, however, schools and public spaces may require that masks be worn to mitigate the spread of the virus, particularly as more virulent and transmittable forms of the virus mutate. Because of the current rise in coronavirus cases in every state, the increase in the younger population affected, and the fact that the delta variant appears to be a virulent strain, we believe it is important to assess the effect of wearing a mask on all ages.

*required

4 Provide the total number of participants (or number of participant records, specimens, etc.) for whom you are seeking IRB approval.

80 - 500. Reviewing the power determination from the single pediatric study of End-Tidal CO2 levels in N95 masks (Goh et all, Scientific Reports, 2019), at least 80 subjects in the pediatric group are desired.

*required

5 Describe what time commitment will be required from each participant, including individual interactions, total time commitment, and long-term follow-up, if any.

The total participant time commitment will be approximately 1/2 hour. Because masks are being worn everywhere with no negative consequences, there will be no follow-up necessary.

*required

6 Describe how potential participants will be identified (e.g., advertising, individuals known to investigator, record review, etc.). Explain how investigator(s) will gain access to this population, as applicable.

Our study includes age groups from 2 to 14 (inclusive) and 18-80 (inclusive), individuals, and families. Researchers will invite those in the local community to participate in the study.

*required

7 Describe the recruitment process; including the setting in which recruitment will take place.

Recruitment of participants will occur via direct communication (word of mouth), emails, and flyers in the community.

Attach recruitment materials (ads, flyers, website postings, recruitment letters, and oral/written scripts), if applicable .

flyer mask CO2.pdf

*required

8 Will participants receive compensation or other incentives (e.g., free services, cash payments, gift certificates, parking, classroom credit, travel reimbursement, etc.) to participate in the research study?

Yes

✓ No

Risks and Benefits

*required

1 Describe all reasonably expected risks, harms, and/or discomforts that may apply to the research. Discuss severity and likelihood of occurrence.

Consider the range of risks - physical, psychological, social, legal, and economic.

There are no expected risks or harms related to this study. Although most participants have recently been required to wear masks in public, some may feel some discomfort in doing so. A new disposable surgical mask, of the same model, in appropriate sizes, will be worn by all participants. It is similar to one that is approved for the surgical theater and worn by all health care providers in an operating room. The nasal cannula, used daily in operating rooms in the US, is not associated with pain or discomfort but will be a new experience for participants.

*required

2 Discuss the steps that will be taken to minimize risks and the likelihood of harm.

Should a child or adult decline participation after masking or wearing a nasal cannula, they may withdraw from the study at any time.

*required

3 Describe the potential benefits that participants may expect as a result of this research study. State if there are no direct benefits to individual participants.

No direct benefits to individual participants.

*required

Discuss any potential indirect benefits to future subjects, science, and society.

The American Association of Pediatrics Covid-19 Guidance for Safe Schools (as of 7/18/21) has recommended:

- All eligible individuals should receive the COVID-19 vaccine.
    - It may become necessary for schools to collect COVID-19 vaccine information of staff and students and for schools to require COVID-19 vaccination for in-person learning.
    - Adequate and timely COVID-19 vaccination resources for the whole school community must be available and accessible.
- **All students older than 2 years and all school staff should wear face masks at school (unless medical or developmental conditions prohibit use).**
    - The AAP recommends universal masking in school at this time for the following reasons:
        - a significant portion of the student population is not eligible for vaccination
        - protection of unvaccinated students from COVID-19 and to reduce transmission
            - lack of a system to monitor vaccine status among students, teachers, and staff
        - potential difficulty in monitoring or enforcing mask policies for those who are not vaccinated; in the absence of schools being able to conduct this monitoring, universal masking is the best and most effective strategy to create consistent
4 messages, expectations, enforcement, and compliance without the added burden of needing to monitor vaccination status
        - possibility of low vaccination uptake within the surrounding school community
        - continued concerns for variants that are more easily spread among children, adolescents, and adults

Masking during the Covid-19 epidemic has been an emotionally and politically charged topic. There are widespread public misconceptions about the benefits and risks of masking, particularly in children. A since retracted JAMA Pediatrics publication claimed that masking was harmful to children due to elevated CO2 levels. As discussed elsewhere in this application, the study was fatally flawed and its conclusions were unsupported by scientific study or any appropriate scientific references. Nonetheless, that study sparked public outrage against mask use in schools and has been forwarded to school boards as "evidence" that masks should not be worn in schools.

This study, the first of its kind, aims to answer whether surgical masks, worn by both adults and children, cause any concerning abnormality in oxygenation or carbon dioxide levels, as measured by noninvasive means. Despite the fact that adults and children have been wearing masks for extended periods of time without low oxygen or high carbon dioxide side effects, there is an appetite for the final answer on this topic. School systems, parents, and the general public can benefit from seeing the results of this study.

*required

Describe how risks to participants are reasonable when compared to the anticipated benefits to participants (if any) and teh importance of the knowledge that may reasonably be expected to result.

Children and adults wear surgical-type masks without harm, as is evidenced by recommendations from the American Academy of Pediatricians. The novel experience of volunteer adults and children wearing a nasal cannula will be outweighed by their general interest in helping answer a sometimes emotionally charged question and providing knowledge to parents, educators, and the public.

Informed Consent

*required

1 From the list below, indicate how consent will be obtained for this study.

Check all that apply.

✓ Written/signed consent by the subject

✓ Written/signed consent (permission) for a minor by a Parent or Legal Guardian

Written/signed consent by a Legally Authorized Representative (for adults incapable of consenting)

Request for waiver of documentation of consent (verbal consent, anonymous surveys, etc.)

Waiver of parental permission

Waiver of consent (consent will not be obtained from subjects)

*required

2 Describe the consent process including where and by whom the subjects will be approached, the plans to ensure the privacy of the subjects and the measures to ensure that subjects understand the nature of the study, its procedures, risks and benefits and that they freely grant their consent.

Upon arrival to the designated area, participants will be instructed on the study, the procedures, any risks or benefits, and given the consent form for signature, for either adult or for a minor by a parent or legal guardian. The subjects/parents of subjects will speak with a medical professional with experience working with medical consent forms, and freely granted consent will be assured. All questions will be addressed. The subjects can choose to avoid answering the inclusion/exclusion screening questions and can avoid joining the study, or withdraw from the study, at any time.

*required

Attach all consent and assent documents here:

Assent Form Mask CO2 study.docx

consent CO2 mask study.docx

Data Collection

Missouri State University is committed to keeping data and information secure. Please review the Missouri Ste University Information Security Policies. Discuss you project with the MSU Information Security Office or your College's IT support staff if you have questions about how to handle your data appropriately.

*required

1 **Statement of Principal Investigator Responsibility for Data**

The principal investigator of this study is responsible for the storage, oversight, and disposal of all data associated with this study. Data will not be disseminated without the explicit approval of the principal investigator, and identifying information associated with the data will not be shared.

✓ By checking this box, all personnel associated with this study understand and agree to the Statement of Principal Investigator Responsibility for Data.

*required

2 How will the data for this study be collected/stored?

Check all that apply.

✓ Electronic Storage Format

On paper

*required

Describe where the data will be stored (e.g., paper forms, flash drives or removable media, desktop or laptop computer, server, research storage area network, external source) and describe the plan to ensure the security and confidentiality of the records (e.g., locked office, locked file cabinet, password-protected computer or files, encrypted data files, database limited to coded data, master list stored in separate location).

3 *At minimum, physical data should always be secured by lock and key when stored. Electronic data should be stored on University secure servers whenever possible (Office 365 or other secure campus server). If data has to be stored off campus, the file should be encrypted and the device password protected. Additionally, any data to be shared outside the University network will require a SUDERS request be filed and approved. See https://mis.missouristate.edu/Central/suders/create*

Data will be stored on an excel spreadsheet on the Missouri State onedrive. The excel spreadsheet will list the subjects only by subject number. No identifying data will be included on the spreadsheet. The study consent forms will be the only location which includes patients' names. These will be kept in a locked cabinet in the Department of Anesthesia.

*required

Describe how data will be disposed of and when disposal will occur.

4 *At minimum, Federal regulations require research records to be retained for at least 3 years after the completion of the research (45 CFR 46). Research that involves identifiable health information is subject to HIPAA regulations, which require records to be retained for at least 6 years after a participant has signed an authorization. Finally, funded research projects may require longer retention periods, you may need to follow the sponsoring agency guidelines.*

Excel spreadsheets, which will not include identifyable data, will be deleted after 3 years.

Consent forms, which include patients names/signatures will be destroyed by shreading after 6 years.

Additional Information

1 Please include any additional information about the study below.

2 Please include any additional documents that aren't covered within the application.
