# Supplementary material for: Physiologic effects of surgical masking in children versus adults
Source: PeerJ. 2023 Jun 16;11:e15474. doi: 10.7717/peerj.15474 (PMC10278594; doi:10.7717/peerj.15474)
Supplement: Supplemental Information 4 [file peerj-11-15474-s004.docx]

**ETCO2**

| **Within Subjects Effects** | | | | | | | | | | | | | | | |
| --- | --- | --- | --- | --- | --- | --- | --- | --- | --- | --- | --- | --- | --- | --- | --- |
| **Cases** | | **Sphericity Correction** | | **Sum of Squares** | | **df** | | **Mean Square** | | **F** | | **p** | | **η²** | |
| ETCO2 Levels |  | None |  | 53.330 | ᵃ | 3.000 | ᵃ | 17.777 | ᵃ | 6.757 | ᵃ | < .001 | ᵃ | 0.025 |  |
|  |  | Greenhouse-Geisser |  | 53.330 |  | 1.945 |  | 27.413 |  | 6.757 |  | 0.002 |  | 0.025 |  |
| ETCO2 Levels ✻ Age_Rev_Group |  | None |  | 5.728 | ᵃ | 3.000 | ᵃ | 1.909 | ᵃ | 0.726 | ᵃ | 0.538 | ᵃ | 0.003 |  |
|  |  | Greenhouse-Geisser |  | 5.728 |  | 1.945 |  | 2.944 |  | 0.726 |  | 0.483 |  | 0.003 |  |
| Residuals |  | None |  | 363.048 |  | 138.000 |  | 2.631 |  |  |  |  |  |  |  |
|  |  | Greenhouse-Geisser |  | 363.048 |  | 89.492 |  | 4.057 |  |  |  |  |  |  |  |
|  | | | | | | | | | | | | | | | |
| *Note.*  Type III Sum of Squares | | | | | | | | | | | | | | | |
| ᵃ Mauchly's test of sphericity indicates that the assumption of sphericity is violated (p < .05). | | | | | | | | | | | | | | | |

| **Between Subjects Effects** | | | | | | | | | | | | | |
| --- | --- | --- | --- | --- | --- | --- | --- | --- | --- | --- | --- | --- | --- |
| **Cases** | | **Sum of Squares** | | **df** | | **Mean Square** | | **F** | | **p** | | **η²** | |
| Age_Rev_Group |  | 6.006 |  | 1 |  | 6.006 |  | 0.160 |  | 0.691 |  | 0.003 |  |
| Residuals |  | 1731.468 |  | 46 |  | 37.641 |  |  |  |  |  |  |  |
|  | | | | | | | | | | | | | |
| *Note.*  Type III Sum of Squares | | | | | | | | | | | | | |

**Descriptives**

| **Descriptives** | | | | | | | | | |
| --- | --- | --- | --- | --- | --- | --- | --- | --- | --- |
| **ETCO2 Levels** | | **Age_Rev_Group** | | **Mean** | | **SD** | | **N** | |
| No Mast - First 5 |  | 1 |  | 34.150 |  | 3.934 |  | 20 |  |
|  |  | 2 |  | 33.400 |  | 3.636 |  | 28 |  |
| Mask - First 5 |  | 1 |  | 35.340 |  | 3.459 |  | 20 |  |
|  |  | 2 |  | 34.779 |  | 3.252 |  | 28 |  |
| Mask - Sec 5 |  | 1 |  | 34.670 |  | 2.542 |  | 20 |  |
|  |  | 2 |  | 34.854 |  | 3.080 |  | 28 |  |
| Mask - Last 5 |  | 1 |  | 35.250 |  | 3.335 |  | 20 |  |
|  |  | 2 |  | 34.943 |  | 3.551 |  | 28 |  |
|  | | | | | | | | | |

**Assumption Checks**

| **Test of Sphericity** | | | | | | | | | | | | | | | |
| --- | --- | --- | --- | --- | --- | --- | --- | --- | --- | --- | --- | --- | --- | --- | --- |
|  | | **Mauchly's W** | | **Approx. Χ²** | | **df** | | **p-value** | | **Greenhouse-Geisser ε** | | **Huynh-Feldt ε** | | **Lower Bound ε** | |
| ETCO2 Levels |  | 0.468 |  | 33.998 |  | 5 |  | < .001 |  | 0.648 |  | 0.677 |  | 0.333 |  |
|  | | | | | | | | | | | | | | | |

**Post Hoc Tests**

| **Post Hoc Comparisons - ETCO2 Levels** | | | | | | | | | | | | | |
| --- | --- | --- | --- | --- | --- | --- | --- | --- | --- | --- | --- | --- | --- |
|  | |  | | **Mean Difference** | | **SE** | | **t** | | **Cohen's d** | | **p_bonf_** | |
| No Mast - First 5 |  | Mask - First 5 |  | -1.284 |  | 0.336 |  | -3.825 |  | -0.552 |  | 0.001 | ** |
|  |  | Mask - Sec 5 |  | -0.987 |  | 0.336 |  | -2.939 |  | -0.424 |  | 0.023 | * |
|  |  | Mask - Last 5 |  | -1.321 |  | 0.336 |  | -3.935 |  | -0.568 |  | < .001 | *** |
| Mask - First 5 |  | Mask - Sec 5 |  | 0.297 |  | 0.336 |  | 0.886 |  | 0.128 |  | 1.000 |  |
|  |  | Mask - Last 5 |  | -0.037 |  | 0.336 |  | -0.111 |  | -0.016 |  | 1.000 |  |
| Mask - Sec 5 |  | Mask - Last 5 |  | -0.335 |  | 0.336 |  | -0.997 |  | -0.144 |  | 1.000 |  |
|  | | | | | | | | | | | | | |
| * p < .05, ** p < .01, *** p < .001 | | | | | | | | | | | | | |
| *Note.*  Cohen's d does not correct for multiple comparisons. | | | | | | | | | | | | | |
| *Note.*  P-value adjusted for comparing a family of 6 | | | | | | | | | | | | | |
| *Note.*  Results are averaged over the levels of: Age_Rev_Group | | | | | | | | | | | | | |

**Marginal Means**

| **Marginal Means - ETCO2 Levels** | | | | | | | | | |
| --- | --- | --- | --- | --- | --- | --- | --- | --- | --- |
|  | | | | **95% CI for Mean Difference** | | | |  | |
| **ETCO2 Levels** | | **Marginal Mean** | | **Lower** | | **Upper** | | **SE** | |
| No Mast - First 5 |  | 33.745 |  | 32.771 |  | 34.720 |  | 0.488 |  |
| Mask - First 5 |  | 35.029 |  | 34.055 |  | 36.004 |  | 0.488 |  |
| Mask - Sec 5 |  | 34.732 |  | 33.757 |  | 35.706 |  | 0.488 |  |
| Mask - Last 5 |  | 35.067 |  | 34.092 |  | 36.041 |  | 0.488 |  |
|  | | | | | | | | | |

| **Marginal Means - Age_Rev_Group** | | | | | | | | | |
| --- | --- | --- | --- | --- | --- | --- | --- | --- | --- |
|  | | | | **95% CI for Mean Difference** | | | |  | |
| **Age_Rev_Group** | | **Marginal Mean** | | **Lower** | | **Upper** | | **SE** | |
| 1 |  | 34.823 |  | 33.553 |  | 36.092 |  | 0.631 |  |
| 2 |  | 34.464 |  | 33.194 |  | 35.733 |  | 0.631 |  |
|  | | | | | | | | | |

**ICO2**

| **Within Subjects Effects** | | | | | | | | | | | | | | | |
| --- | --- | --- | --- | --- | --- | --- | --- | --- | --- | --- | --- | --- | --- | --- | --- |
| **Cases** | | **Sphericity Correction** | | **Sum of Squares** | | **df** | | **Mean Square** | | **F** | | **p** | | **η²** | |
| ICO2 Levels |  | None |  | 306.478 | ᵃ | 3.000 | ᵃ | 102.159 | ᵃ | 63.362 | ᵃ | < .001 | ᵃ | 0.248 |  |
|  |  | Huynh-Feldt |  | 306.478 |  | 2.670 |  | 114.779 |  | 63.362 |  | < .001 |  | 0.248 |  |
| ICO2 Levels ✻ Age_Rev_Group |  | None |  | 45.421 | ᵃ | 3.000 | ᵃ | 15.140 | ᵃ | 9.391 | ᵃ | < .001 | ᵃ | 0.037 |  |
|  |  | Huynh-Feldt |  | 45.421 |  | 2.670 |  | 17.011 |  | 9.391 |  | < .001 |  | 0.037 |  |
| Residuals |  | None |  | 222.498 |  | 138.000 |  | 1.612 |  |  |  |  |  |  |  |
|  |  | Huynh-Feldt |  | 222.498 |  | 122.827 |  | 1.811 |  |  |  |  |  |  |  |
|  | | | | | | | | | | | | | | | |
| *Note.*  Type III Sum of Squares | | | | | | | | | | | | | | | |
| ᵃ Mauchly's test of sphericity indicates that the assumption of sphericity is violated (p < .05). | | | | | | | | | | | | | | | |

| **Between Subjects Effects** | | | | | | | | | | | | | |
| --- | --- | --- | --- | --- | --- | --- | --- | --- | --- | --- | --- | --- | --- |
| **Cases** | | **Sum of Squares** | | **df** | | **Mean Square** | | **F** | | **p** | | **η²** | |
| Age_Rev_Group |  | 199.893 |  | 1 |  | 199.893 |  | 19.922 |  | < .001 |  | 0.162 |  |
| Residuals |  | 461.552 |  | 46 |  | 10.034 |  |  |  |  |  |  |  |
|  | | | | | | | | | | | | | |
| *Note.*  Type III Sum of Squares | | | | | | | | | | | | | |

**Descriptives**

| **Descriptives** | | | | | | | | | |
| --- | --- | --- | --- | --- | --- | --- | --- | --- | --- |
| **ICO2 Levels** | | **Age_Rev_Group** | | **Mean** | | **SD** | | **N** | |
| No Mask - first 5 |  | 1 |  | 3.560 |  | 1.239 |  | 20 |  |
|  |  | 2 |  | 3.107 |  | 1.694 |  | 28 |  |
| Mask - First 5 |  | 1 |  | 7.140 |  | 2.279 |  | 20 |  |
|  |  | 2 |  | 4.786 |  | 1.844 |  | 28 |  |
| Mask - Sec 5 |  | 1 |  | 7.880 |  | 2.344 |  | 20 |  |
|  |  | 2 |  | 4.750 |  | 1.807 |  | 28 |  |
| Mask - Last 5 |  | 1 |  | 7.670 |  | 2.272 |  | 20 |  |
|  |  | 2 |  | 5.329 |  | 1.888 |  | 28 |  |
|  | | | | | | | | | |

**Assumption Checks**

| **Test of Sphericity** | | | | | | | | | | | | | | | |
| --- | --- | --- | --- | --- | --- | --- | --- | --- | --- | --- | --- | --- | --- | --- | --- |
|  | | **Mauchly's W** | | **Approx. Χ²** | | **df** | | **p-value** | | **Greenhouse-Geisser ε** | | **Huynh-Feldt ε** | | **Lower Bound ε** | |
| ICO2 Levels |  | 0.724 |  | 14.445 |  | 5 |  | 0.013 |  | 0.838 |  | 0.890 |  | 0.333 |  |
|  | | | | | | | | | | | | | | | |

**Post Hoc Tests**

| **Post Hoc Comparisons - ICO2 Levels** | | | | | | | | | | | | | |
| --- | --- | --- | --- | --- | --- | --- | --- | --- | --- | --- | --- | --- | --- |
|  | |  | | **Mean Difference** | | **SE** | | **t** | | **Cohen's d** | | **p_bonf_** | |
| No Mask - first 5 |  | Mask - First 5 |  | -2.629 |  | 0.263 |  | -10.002 |  | -1.444 |  | < .001 | *** |
|  |  | Mask - Sec 5 |  | -2.981 |  | 0.263 |  | -11.342 |  | -1.637 |  | < .001 | *** |
|  |  | Mask - Last 5 |  | -3.166 |  | 0.263 |  | -12.043 |  | -1.738 |  | < .001 | *** |
| Mask - First 5 |  | Mask - Sec 5 |  | -0.352 |  | 0.263 |  | -1.340 |  | -0.193 |  | 1.000 |  |
|  |  | Mask - Last 5 |  | -0.536 |  | 0.263 |  | -2.041 |  | -0.295 |  | 0.259 |  |
| Mask - Sec 5 |  | Mask - Last 5 |  | -0.184 |  | 0.263 |  | -0.701 |  | -0.101 |  | 1.000 |  |
|  | | | | | | | | | | | | | |
| *** p < .001 | | | | | | | | | | | | | |
| *Note.*  Cohen's d does not correct for multiple comparisons. | | | | | | | | | | | | | |
| *Note.*  P-value adjusted for comparing a family of 6 | | | | | | | | | | | | | |
| *Note.*  Results are averaged over the levels of: Age_Rev_Group | | | | | | | | | | | | | |

| **Post Hoc Comparisons - Age_Rev_Group** | | | | | | | | | | | | | |
| --- | --- | --- | --- | --- | --- | --- | --- | --- | --- | --- | --- | --- | --- |
|  | |  | | **Mean Difference** | | **SE** | | **t** | | **Cohen's d** | | **p_bonf_** | |
| 1 |  | 2 |  | 2.070 |  | 0.464 |  | 4.463 |  | 0.644 |  | < .001 | *** |
|  | | | | | | | | | | | | | |
| *** p < .001 | | | | | | | | | | | | | |
| *Note.*  Cohen's d does not correct for multiple comparisons. | | | | | | | | | | | | | |
| *Note.*  Results are averaged over the levels of: ICO2 Levels | | | | | | | | | | | | | |

| **Post Hoc Comparisons - Age_Rev_Group ✻ ICO2 Levels** | | | | | | | | | | | |
| --- | --- | --- | --- | --- | --- | --- | --- | --- | --- | --- | --- |
|  | |  | | **Mean Difference** | | **SE** | | **t** | | **p_bonf_** | |
| 1, No Mask - first 5 |  | 2, No Mask - first 5 |  | 0.453 |  | 0.564 |  | 0.802 |  | 1.000 |  |
|  |  | 1, Mask - First 5 |  | -3.580 |  | 0.402 |  | -8.916 |  | < .001 | *** |
|  |  | 2, Mask - First 5 |  | -1.226 |  | 0.564 |  | -2.171 |  | 0.908 |  |
|  |  | 1, Mask - Sec 5 |  | -4.320 |  | 0.402 |  | -10.759 |  | < .001 | *** |
|  |  | 2, Mask - Sec 5 |  | -1.190 |  | 0.564 |  | -2.108 |  | 1.000 |  |
|  |  | 1, Mask - Last 5 |  | -4.110 |  | 0.402 |  | -10.236 |  | < .001 | *** |
|  |  | 2, Mask - Last 5 |  | -1.769 |  | 0.564 |  | -3.133 |  | 0.065 |  |
| 2, No Mask - first 5 |  | 1, Mask - First 5 |  | -4.033 |  | 0.564 |  | -7.144 |  | < .001 | *** |
|  |  | 2, Mask - First 5 |  | -1.679 |  | 0.339 |  | -4.946 |  | < .001 | *** |
|  |  | 1, Mask - Sec 5 |  | -4.773 |  | 0.564 |  | -8.455 |  | < .001 | *** |
|  |  | 2, Mask - Sec 5 |  | -1.643 |  | 0.339 |  | -4.841 |  | < .001 | *** |
|  |  | 1, Mask - Last 5 |  | -4.563 |  | 0.564 |  | -8.083 |  | < .001 | *** |
|  |  | 2, Mask - Last 5 |  | -2.221 |  | 0.339 |  | -6.546 |  | < .001 | *** |
| 1, Mask - First 5 |  | 2, Mask - First 5 |  | 2.354 |  | 0.564 |  | 4.171 |  | 0.002 | ** |
|  |  | 1, Mask - Sec 5 |  | -0.740 |  | 0.402 |  | -1.843 |  | 1.000 |  |
|  |  | 2, Mask - Sec 5 |  | 2.390 |  | 0.564 |  | 4.234 |  | 0.001 | ** |
|  |  | 1, Mask - Last 5 |  | -0.530 |  | 0.402 |  | -1.320 |  | 1.000 |  |
|  |  | 2, Mask - Last 5 |  | 1.811 |  | 0.564 |  | 3.209 |  | 0.051 |  |
| 2, Mask - First 5 |  | 1, Mask - Sec 5 |  | -3.094 |  | 0.564 |  | -5.481 |  | < .001 | *** |
|  |  | 2, Mask - Sec 5 |  | 0.036 |  | 0.339 |  | 0.105 |  | 1.000 |  |
|  |  | 1, Mask - Last 5 |  | -2.884 |  | 0.564 |  | -5.109 |  | < .001 | *** |
|  |  | 2, Mask - Last 5 |  | -0.543 |  | 0.339 |  | -1.600 |  | 1.000 |  |
| 1, Mask - Sec 5 |  | 2, Mask - Sec 5 |  | 3.130 |  | 0.564 |  | 5.545 |  | < .001 | *** |
|  |  | 1, Mask - Last 5 |  | 0.210 |  | 0.402 |  | 0.523 |  | 1.000 |  |
|  |  | 2, Mask - Last 5 |  | 2.551 |  | 0.564 |  | 4.520 |  | < .001 | *** |
| 2, Mask - Sec 5 |  | 1, Mask - Last 5 |  | -2.920 |  | 0.564 |  | -5.173 |  | < .001 | *** |
|  |  | 2, Mask - Last 5 |  | -0.579 |  | 0.339 |  | -1.705 |  | 1.000 |  |
| 1, Mask - Last 5 |  | 2, Mask - Last 5 |  | 2.341 |  | 0.564 |  | 4.148 |  | 0.002 | ** |
|  | | | | | | | | | | | |
| * p < .05, ** p < .01, *** p < .001 | | | | | | | | | | | |
| *Note.*  P-value adjusted for comparing a family of 28 | | | | | | | | | | | |

**Marginal Means**

| **Marginal Means - ICO2 Levels** | | | | | | | | | |
| --- | --- | --- | --- | --- | --- | --- | --- | --- | --- |
|  | | | | **95% CI for Mean Difference** | | | |  | |
| **ICO2 Levels** | | **Marginal Mean** | | **Lower** | | **Upper** | | **SE** | |
| No Mask - first 5 |  | 3.161 |  | 2.606 |  | 3.716 |  | 0.280 |  |
| Mask - First 5 |  | 5.790 |  | 5.235 |  | 6.345 |  | 0.280 |  |
| Mask - Sec 5 |  | 6.143 |  | 5.587 |  | 6.698 |  | 0.280 |  |
| Mask - Last 5 |  | 6.327 |  | 5.772 |  | 6.882 |  | 0.280 |  |
|  | | | | | | | | | |

| **Marginal Means - Age_Rev_Group** | | | | | | | | | |
| --- | --- | --- | --- | --- | --- | --- | --- | --- | --- |
|  | | | | **95% CI for Mean Difference** | | | |  | |
| **Age_Rev_Group** | | **Marginal Mean** | | **Lower** | | **Upper** | | **SE** | |
| 1 |  | 6.390 |  | 5.735 |  | 7.045 |  | 0.326 |  |
| 2 |  | 4.320 |  | 3.665 |  | 4.976 |  | 0.326 |  |
|  | | | | | | | | | |

**RR**

| **Within Subjects Effects** | | | | | | | | | | | | | |
| --- | --- | --- | --- | --- | --- | --- | --- | --- | --- | --- | --- | --- | --- |
| **Cases** | | **Sum of Squares** | | **df** | | **Mean Square** | | **F** | | **p** | | **η²** | |
| RR Levels |  | 59.891 | ᵃ | 3 | ᵃ | 19.964 | ᵃ | 3.925 | ᵃ | 0.010 | ᵃ | 0.015 |  |
| RR Levels ✻ Age_Rev_Group |  | 66.961 | ᵃ | 3 | ᵃ | 22.320 | ᵃ | 4.388 | ᵃ | 0.006 | ᵃ | 0.017 |  |
| Residuals |  | 701.975 |  | 138 |  | 5.087 |  |  |  |  |  |  |  |
|  | | | | | | | | | | | | | |
| *Note.*  Type III Sum of Squares | | | | | | | | | | | | | |
| ᵃ Mauchly's test of sphericity indicates that the assumption of sphericity is violated (p < .05). | | | | | | | | | | | | | |

| **Between Subjects Effects** | | | | | | | | | | | | | |
| --- | --- | --- | --- | --- | --- | --- | --- | --- | --- | --- | --- | --- | --- |
| **Cases** | | **Sum of Squares** | | **df** | | **Mean Square** | | **F** | | **p** | | **η²** | |
| Age_Rev_Group |  | 985.321 |  | 1 |  | 985.321 |  | 20.980 |  | < .001 |  | 0.248 |  |
| Residuals |  | 2160.378 |  | 46 |  | 46.965 |  |  |  |  |  |  |  |
|  | | | | | | | | | | | | | |
| *Note.*  Type III Sum of Squares | | | | | | | | | | | | | |

**Descriptives**

| **Descriptives** | | | | | | | | | |
| --- | --- | --- | --- | --- | --- | --- | --- | --- | --- |
| **RR Levels** | | **Age_Rev_Group** | | **Mean** | | **SD** | | **N** | |
| No Mask - First 5 |  | 1 |  | 18.480 |  | 3.516 |  | 20 |  |
|  |  | 2 |  | 15.693 |  | 2.712 |  | 28 |  |
| Mask - First 5 |  | 1 |  | 20.800 |  | 4.618 |  | 20 |  |
|  |  | 2 |  | 15.500 |  | 3.048 |  | 28 |  |
| Mask - Sec 5 |  | 1 |  | 21.630 |  | 4.753 |  | 20 |  |
|  |  | 2 |  | 15.657 |  | 4.409 |  | 28 |  |
| Mask - Last 5 |  | 1 |  | 19.970 |  | 5.173 |  | 20 |  |
|  |  | 2 |  | 15.650 |  | 3.390 |  | 28 |  |
|  | | | | | | | | | |

**Assumption Checks**

| **Test of Sphericity** | | | | | | | | | | | | | | | |
| --- | --- | --- | --- | --- | --- | --- | --- | --- | --- | --- | --- | --- | --- | --- | --- |
|  | | **Mauchly's W** | | **Approx. Χ²** | | **df** | | **p-value** | | **Greenhouse-Geisser ε** | | **Huynh-Feldt ε** | | **Lower Bound ε** | |
| RR Levels |  | 0.777 |  | 11.289 |  | 5 |  | 0.046 |  | 0.885 |  | 0.945 |  | 0.333 |  |
|  | | | | | | | | | | | | | | | |

**Post Hoc Tests**

| **Post Hoc Comparisons - RR Levels** | | | | | | | | | | | | | |
| --- | --- | --- | --- | --- | --- | --- | --- | --- | --- | --- | --- | --- | --- |
|  | |  | | **Mean Difference** | | **SE** | | **t** | | **Cohen's d** | | **p_bonf_** | |
| No Mask - First 5 |  | Mask - First 5 |  | -1.064 |  | 0.467 |  | -2.278 |  | -0.329 |  | 0.146 |  |
|  |  | Mask - Sec 5 |  | -1.557 |  | 0.467 |  | -3.335 |  | -0.481 |  | 0.007 | ** |
|  |  | Mask - Last 5 |  | -0.724 |  | 0.467 |  | -1.550 |  | -0.224 |  | 0.741 |  |
| Mask - First 5 |  | Mask - Sec 5 |  | -0.494 |  | 0.467 |  | -1.057 |  | -0.153 |  | 1.000 |  |
|  |  | Mask - Last 5 |  | 0.340 |  | 0.467 |  | 0.728 |  | 0.105 |  | 1.000 |  |
| Mask - Sec 5 |  | Mask - Last 5 |  | 0.834 |  | 0.467 |  | 1.785 |  | 0.258 |  | 0.458 |  |
|  | | | | | | | | | | | | | |
| ** p < .01 | | | | | | | | | | | | | |
| *Note.*  Cohen's d does not correct for multiple comparisons. | | | | | | | | | | | | | |
| *Note.*  P-value adjusted for comparing a family of 6 | | | | | | | | | | | | | |
| *Note.*  Results are averaged over the levels of: Age_Rev_Group | | | | | | | | | | | | | |

| **Post Hoc Comparisons - Age_Rev_Group** | | | | | | | | | | | | | |
| --- | --- | --- | --- | --- | --- | --- | --- | --- | --- | --- | --- | --- | --- |
|  | |  | | **Mean Difference** | | **SE** | | **t** | | **Cohen's d** | | **p_bonf_** | |
| 1 |  | 2 |  | 4.595 |  | 1.003 |  | 4.580 |  | 0.661 |  | < .001 | *** |
|  | | | | | | | | | | | | | |
| *** p < .001 | | | | | | | | | | | | | |
| *Note.*  Cohen's d does not correct for multiple comparisons. | | | | | | | | | | | | | |
| *Note.*  Results are averaged over the levels of: RR Levels | | | | | | | | | | | | | |

| **Post Hoc Comparisons - Age_Rev_Group ✻ RR Levels** | | | | | | | | | | | |
| --- | --- | --- | --- | --- | --- | --- | --- | --- | --- | --- | --- |
|  | |  | | **Mean Difference** | | **SE** | | **t** | | **p_bonf_** | |
| 1, No Mask - First 5 |  | 2, No Mask - First 5 |  | 2.787 |  | 1.155 |  | 2.414 |  | 0.508 |  |
|  |  | 1, Mask - First 5 |  | -2.320 |  | 0.713 |  | -3.253 |  | 0.040 | * |
|  |  | 2, Mask - First 5 |  | 2.980 |  | 1.155 |  | 2.581 |  | 0.329 |  |
|  |  | 1, Mask - Sec 5 |  | -3.150 |  | 0.713 |  | -4.417 |  | < .001 | *** |
|  |  | 2, Mask - Sec 5 |  | 2.823 |  | 1.155 |  | 2.445 |  | 0.469 |  |
|  |  | 1, Mask - Last 5 |  | -1.490 |  | 0.713 |  | -2.089 |  | 1.000 |  |
|  |  | 2, Mask - Last 5 |  | 2.830 |  | 1.155 |  | 2.451 |  | 0.462 |  |
| 2, No Mask - First 5 |  | 1, Mask - First 5 |  | -5.107 |  | 1.155 |  | -4.423 |  | < .001 | *** |
|  |  | 2, Mask - First 5 |  | 0.193 |  | 0.603 |  | 0.320 |  | 1.000 |  |
|  |  | 1, Mask - Sec 5 |  | -5.937 |  | 1.155 |  | -5.142 |  | < .001 | *** |
|  |  | 2, Mask - Sec 5 |  | 0.036 |  | 0.603 |  | 0.059 |  | 1.000 |  |
|  |  | 1, Mask - Last 5 |  | -4.277 |  | 1.155 |  | -3.704 |  | 0.011 | * |
|  |  | 2, Mask - Last 5 |  | 0.043 |  | 0.603 |  | 0.071 |  | 1.000 |  |
| 1, Mask - First 5 |  | 2, Mask - First 5 |  | 5.300 |  | 1.155 |  | 4.590 |  | < .001 | *** |
|  |  | 1, Mask - Sec 5 |  | -0.830 |  | 0.713 |  | -1.164 |  | 1.000 |  |
|  |  | 2, Mask - Sec 5 |  | 5.143 |  | 1.155 |  | 4.454 |  | < .001 | *** |
|  |  | 1, Mask - Last 5 |  | 0.830 |  | 0.713 |  | 1.164 |  | 1.000 |  |
|  |  | 2, Mask - Last 5 |  | 5.150 |  | 1.155 |  | 4.460 |  | < .001 | *** |
| 2, Mask - First 5 |  | 1, Mask - Sec 5 |  | -6.130 |  | 1.155 |  | -5.309 |  | < .001 | *** |
|  |  | 2, Mask - Sec 5 |  | -0.157 |  | 0.603 |  | -0.261 |  | 1.000 |  |
|  |  | 1, Mask - Last 5 |  | -4.470 |  | 1.155 |  | -3.871 |  | 0.006 | ** |
|  |  | 2, Mask - Last 5 |  | -0.150 |  | 0.603 |  | -0.249 |  | 1.000 |  |
| 1, Mask - Sec 5 |  | 2, Mask - Sec 5 |  | 5.973 |  | 1.155 |  | 5.173 |  | < .001 | *** |
|  |  | 1, Mask - Last 5 |  | 1.660 |  | 0.713 |  | 2.327 |  | 0.599 |  |
|  |  | 2, Mask - Last 5 |  | 5.980 |  | 1.155 |  | 5.179 |  | < .001 | *** |
| 2, Mask - Sec 5 |  | 1, Mask - Last 5 |  | -4.313 |  | 1.155 |  | -3.735 |  | 0.010 | ** |
|  |  | 2, Mask - Last 5 |  | 0.007 |  | 0.603 |  | 0.012 |  | 1.000 |  |
| 1, Mask - Last 5 |  | 2, Mask - Last 5 |  | 4.320 |  | 1.155 |  | 3.741 |  | 0.010 | ** |
|  | | | | | | | | | | | |
| * p < .05, ** p < .01, *** p < .001 | | | | | | | | | | | |
| *Note.*  P-value adjusted for comparing a family of 28 | | | | | | | | | | | |

**Marginal Means**

| **Marginal Means - RR Levels** | | | | | | | | | |
| --- | --- | --- | --- | --- | --- | --- | --- | --- | --- |
|  | | | | **95% CI for Mean Difference** | | | |  | |
| **RR Levels** | | **Marginal Mean** | | **Lower** | | **Upper** | | **SE** | |
| No Mask - First 5 |  | 16.704 |  | 15.566 |  | 17.841 |  | 0.571 |  |
| Mask - First 5 |  | 17.767 |  | 16.630 |  | 18.904 |  | 0.571 |  |
| Mask - Sec 5 |  | 18.261 |  | 17.124 |  | 19.398 |  | 0.571 |  |
| Mask - Last 5 |  | 17.427 |  | 16.290 |  | 18.564 |  | 0.571 |  |
|  | | | | | | | | | |

| **Marginal Means - Age_Rev_Group** | | | | | | | | | |
| --- | --- | --- | --- | --- | --- | --- | --- | --- | --- |
|  | | | | **95% CI for Mean Difference** | | | |  | |
| **Age_Rev_Group** | | **Marginal Mean** | | **Lower** | | **Upper** | | **SE** | |
| 1 |  | 19.837 |  | 18.419 |  | 21.255 |  | 0.704 |  |
| 2 |  | 15.242 |  | 13.824 |  | 16.660 |  | 0.704 |  |
|  | | | | | | | | | |

**SpO2**

| **Within Subjects Effects** | | | | | | | | | | | | | |
| --- | --- | --- | --- | --- | --- | --- | --- | --- | --- | --- | --- | --- | --- |
| **Cases** | | **Sum of Squares** | | **df** | | **Mean Square** | | **F** | | **p** | | **η²** | |
| SpO2 Levels |  | 2.484 | ᵃ | 3 | ᵃ | 0.828 | ᵃ | 1.734 | ᵃ | 0.163 | ᵃ | 0.011 |  |
| SpO2 Levels ✻ Age_Rev_Group |  | 5.224 | ᵃ | 3 | ᵃ | 1.741 | ᵃ | 3.646 | ᵃ | 0.014 | ᵃ | 0.024 |  |
| Residuals |  | 65.908 |  | 138 |  | 0.478 |  |  |  |  |  |  |  |
|  | | | | | | | | | | | | | |
| *Note.*  Type III Sum of Squares | | | | | | | | | | | | | |
| ᵃ Mauchly's test of sphericity indicates that the assumption of sphericity is violated (p < .05). | | | | | | | | | | | | | |

| **Between Subjects Effects** | | | | | | | | | | | | | |
| --- | --- | --- | --- | --- | --- | --- | --- | --- | --- | --- | --- | --- | --- |
| **Cases** | | **Sum of Squares** | | **df** | | **Mean Square** | | **F** | | **p** | | **η²** | |
| Age_Rev_Group |  | 0.924 |  | 1 |  | 0.924 |  | 0.298 |  | 0.588 |  | 0.004 |  |
| Residuals |  | 142.675 |  | 46 |  | 3.102 |  |  |  |  |  |  |  |
|  | | | | | | | | | | | | | |
| *Note.*  Type III Sum of Squares | | | | | | | | | | | | | |

**Descriptives**

| **Descriptives** | | | | | | | | | |
| --- | --- | --- | --- | --- | --- | --- | --- | --- | --- |
| **SpO2 Levels** | | **Age_Rev_Group** | | **Mean** | | **SD** | | **N** | |
| No Mask - First 5 |  | 1 |  | 97.400 |  | 1.342 |  | 20 |  |
|  |  | 2 |  | 98.086 |  | 0.947 |  | 28 |  |
| Mask - First 5 |  | 1 |  | 98.120 |  | 0.946 |  | 20 |  |
|  |  | 2 |  | 97.914 |  | 1.276 |  | 28 |  |
| Mask - Sec 5 |  | 1 |  | 98.040 |  | 0.807 |  | 20 |  |
|  |  | 2 |  | 98.007 |  | 1.062 |  | 28 |  |
| Mask - Last 5 |  | 1 |  | 97.820 |  | 1.036 |  | 20 |  |
|  |  | 2 |  | 97.936 |  | 0.981 |  | 28 |  |
|  | | | | | | | | | |

**Assumption Checks**

| **Test of Sphericity** | | | | | | | | | | | | | | | |
| --- | --- | --- | --- | --- | --- | --- | --- | --- | --- | --- | --- | --- | --- | --- | --- |
|  | | **Mauchly's W** | | **Approx. Χ²** | | **df** | | **p-value** | | **Greenhouse-Geisser ε** | | **Huynh-Feldt ε** | | **Lower Bound ε** | |
| SpO2 Levels |  | 0.727 |  | 14.265 |  | 5 |  | 0.014 |  | 0.849 |  | 0.903 |  | 0.333 |  |
|  | | | | | | | | | | | | | | | |

**Marginal Means**

| **Marginal Means - SpO2 Levels** | | | | | | | | | |
| --- | --- | --- | --- | --- | --- | --- | --- | --- | --- |
|  | | | | **95% CI for Mean Difference** | | | |  | |
| **SpO2 Levels** | | **Marginal Mean** | | **Lower** | | **Upper** | | **SE** | |
| No Mask - First 5 |  | 97.755 |  | 97.448 |  | 98.061 |  | 0.154 |  |
| Mask - First 5 |  | 98.029 |  | 97.722 |  | 98.335 |  | 0.154 |  |
| Mask - Sec 5 |  | 98.035 |  | 97.729 |  | 98.342 |  | 0.154 |  |
| Mask - Last 5 |  | 97.890 |  | 97.583 |  | 98.196 |  | 0.154 |  |
|  | | | | | | | | | |

| **Marginal Means - Age_Rev_Group** | | | | | | | | | |
| --- | --- | --- | --- | --- | --- | --- | --- | --- | --- |
|  | | | | **95% CI for Mean Difference** | | | |  | |
| **Age_Rev_Group** | | **Marginal Mean** | | **Lower** | | **Upper** | | **SE** | |
| 1 |  | 97.857 |  | 97.492 |  | 98.221 |  | 0.181 |  |
| 2 |  | 97.997 |  | 97.633 |  | 98.362 |  | 0.181 |  |
|  | | | | | | | | | |

**Post Hoc Tests**

| **Post Hoc Comparisons - Age_Rev_Group ✻ SpO2 Levels** | | | | | | | | | | | | |
| --- | --- | --- | --- | --- | --- | --- | --- | --- | --- | --- | --- | --- |
|  | |  | | **Mean Difference** | | | **SE** | | **t** | | **p_bonf_** | |
| 1, No Mask - First 5 |  | 2, No Mask - First 5 |  | -0.686 | |  | 0.312 |  | -2.200 |  | 0.849 |  |
|  |  | 1, Mask - First 5 |  | -0.720 | |  | 0.219 |  | -3.295 |  | 0.035 |  |
|  |  | 2, Mask - First 5 |  | -0.514 | |  | 0.312 |  | -1.650 |  | 1.000 |  |
|  |  | 1, Mask - Sec 5 |  | -0.640 | |  | 0.219 |  | -2.929 |  | 0.112 |  |
|  |  | 2, Mask - Sec 5 |  | -0.607 | |  | 0.312 |  | -1.948 |  | 1.000 |  |
|  |  | 1, Mask - Last 5 |  | -0.420 | |  | 0.219 |  | -1.922 |  | 1.000 |  |
|  |  | 2, Mask - Last 5 |  | -0.536 | |  | 0.312 |  | -1.719 |  | 1.000 |  |
| 2, No Mask - First 5 |  | 1, Mask - First 5 |  | -0.034 | |  | 0.312 |  | -0.110 |  | 1.000 |  |
|  |  | 2, Mask - First 5 |  | 0.171 | |  | 0.185 |  | 0.928 |  | 1.000 |  |
|  |  | 1, Mask - Sec 5 |  | 0.046 | |  | 0.312 |  | 0.147 |  | 1.000 |  |
|  |  | 2, Mask - Sec 5 |  | 0.079 | |  | 0.185 |  | 0.425 |  | 1.000 |  |
|  |  | 1, Mask - Last 5 |  | 0.266 | |  | 0.312 |  | 0.852 |  | 1.000 |  |
|  |  | 2, Mask - Last 5 |  | 0.150 | |  | 0.185 |  | 0.812 |  | 1.000 |  |
| 1, Mask - First 5 |  | 2, Mask - First 5 |  | 0.206 | |  | 0.312 |  | 0.660 |  | 1.000 |  |
|  |  | 1, Mask - Sec 5 |  | 0.080 | |  | 0.219 |  | 0.366 |  | 1.000 |  |
|  |  | 2, Mask - Sec 5 |  | 0.113 | |  | 0.312 |  | 0.362 |  | 1.000 |  |
|  |  | 1, Mask - Last 5 |  | 0.300 | |  | 0.219 |  | 1.373 |  | 1.000 |  |
|  |  | 2, Mask - Last 5 |  | 0.184 | |  | 0.312 |  | 0.591 |  | 1.000 |  |
| 2, Mask - First 5 |  | 1, Mask - Sec 5 |  | -0.126 | |  | 0.312 |  | -0.403 |  | 1.000 |  |
|  |  | 2, Mask - Sec 5 |  | -0.093 | |  | 0.185 |  | -0.503 |  | 1.000 |  |
|  |  | 1, Mask - Last 5 |  | 0.094 | |  | 0.312 |  | 0.302 |  | 1.000 |  |
|  |  | 2, Mask - Last 5 |  | -0.021 | |  | 0.185 |  | -0.116 |  | 1.000 |  |
| 1, Mask - Sec 5 |  | 2, Mask - Sec 5 |  | 0.033 | |  | 0.312 |  | 0.105 |  | 1.000 |  |
|  |  | 1, Mask - Last 5 |  | 0.220 | |  | 0.219 |  | 1.007 |  | 1.000 |  |
|  |  | 2, Mask - Last 5 |  | 0.104 | |  | 0.312 |  | 0.335 |  | 1.000 |  |
| 2, Mask - Sec 5 |  | 1, Mask - Last 5 |  | 0.187 | |  | 0.312 |  | 0.600 |  | 1.000 |  |
|  |  | 2, Mask - Last 5 |  | 0.071 | |  | 0.185 |  | 0.387 |  | 1.000 |  |
| 1, Mask - Last 5 |  | 2, Mask - Last 5 |  | -0.116 | |  | 0.312 |  | -0.371 |  | 1.000 |  |
|  | | | | | | | | | | | | |
| *Note.*  P-value adjusted for comparing a family of 28 | | | | |  |  |  |  |  |  |  |  |

| *Post Hoc Comparisons for Adult and Pediatric Patients.* | | | | |  |
| --- | --- | --- | --- | --- | --- |
| *Mask Level Comparisons* |  | Adult Patients  *p_bonf_* | Pediatric Patients  (Whole Sample)  *p_bonf_* | Pediatric Patients  (< 7 Years Old)  *p_bonf_* | |
| End-tidal (ETCO2) |  |  |  |  | |
| No Mask - First 5 | Mask - First 5 | <.001*** | < .001*** | .061 | |
|  | Mask - Sec 5 | < .001*** | .009** | 1.00 | |
|  | Mask - Last 5 | < .001*** | < .001*** | .102 | |
| Mask - First 5 | Mask - Sec 5 | 1.00 | 1.00 | .838 | |
|  | Mask - Last 5 | .155 | 1.00 | 1.00 | |
| Mask - Sec 5 | Mask - Last 5 | .860 | 1.00 | 1.00 | |
| ∆Inspired Carbon Dioxide (ICO2) |  |  |  |  | |
| No Mask – Mask First 5 | No Mask - Mask Sec 5 | 1.00 | 1.00 | 1.00 | |
|  | No Mask - Mask Last 5 | 1.00 | 1.00 | 1.00 | |
|  | Mask First 5 - Mask Sec 5 | < .001*** | < .001*** | < .001*** | |
|  | Mask First 5 – Mask Last 5 | < .001*** | < .001*** | < .001*** | |
|  | Mask Sec 5 – Mask Last 5 | < .001*** | < .001*** | < .001*** | |
| No Mask - Mask Sec 5 | No Mask - Mask Last 5 | 1.00 | 1.00 | 1.00 | |
|  | Mask First 5 - Mask Sec 5 | < .001*** | < .001*** | < .001*** | |
|  | Mask First 5 – Mask Last 5 | < .001*** | < .001*** | < .001*** | |
|  | Mask Sec 5 – Mask Last 5 | < .001*** | < .001*** | < .001*** | |
| No Mask - Mask Last 5 | Mask First 5 - Mask Sec 5 | < .001*** | < .001*** | < .001*** | |
|  | Mask First 5 – Mask Last 5 | < .001*** | < .001*** | < .001*** | |
|  | Mask Sec 5 – Mask Last 5 | < .001*** | < .001*** | < .001*** | |
| Mask First 5 - Mask Sec 5 | Mask First 5 – Mask Last 5 | 1.00 | 1.00 | 1.00 | |
|  | Mask Sec 5 – Mask Last 5 | 1.00 | 1.00 | .826 | |
| Mask First 5 – Mask Last 5 | Mask Sec 5 – Mask Last 5 | 1.00 | 1.00 | 1.00 | |
| Respiratory Rate (RR) |  |  |  |  | |
| No Mask - First 5 | Mask - First 5 | NA | NA | 0.011** | |
|  | Mask - Sec 5 | NA | NA | < .001*** | |
|  | Mask - Last 5 | NA | NA | 0.241 | |
| Mask - First 5 | Mask - Sec 5 | NA | NA | 1.000 | |
|  | Mask - Last 5 | NA | NA | 1.000 | |
| Mask - Sec 5 | Mask - Last 5 | NA | NA | 0.137 | |
| Heart Rate (HR) |  |  |  |  | |
| No Mask - First 5 | Mask - First 5 | NA | NA | 0.649 | |
|  | Mask - Sec 5 | NA | NA | 0.017* | |
|  | Mask - Last 5 | NA | NA | 0.297 | |
| Mask - First 5 | Mask - Sec 5 | NA | NA | 0.829 | |
|  | Mask - Last 5 | NA | NA | 1.000 | |
| Mask - Sec 5 | Mask - Last 5 | NA | NA | 1.000 | |

*Note – Post hoc comparisons should be taken with caution for the pediatric subgroup (< 7 years old) as the ANOVAs for ETCO2 and heart rate was not significant after controlling for a type I error*
